# Supplementary material for: Efficacy of JAK1/2 inhibition in murine myeloproliferative neoplasms is not mediated by targeting oncogenic signaling
Source: Nat Commun. 2025 May 24;16:4833. doi: 10.1038/s41467-025-60019-6 (PMC12103521; doi:10.1038/s41467-025-60019-6)
Supplement: Supplementary file 1 — Supplementary Information [file 41467_2025_60019_MOESM1_ESM.docx]

**Efficacy of JAK1/2 inhibition in murine myeloproliferative neoplasms is not mediated by targeting oncogenic signaling**

Sivahari Prasad Gorantla^1,2#^, Michael Rassner^1,3#^, Kirstyn Anne Crossley^1^, Tony Andreas Müller^1^, Teresa Poggio^1^, Shifa Khaja Saleem^1^, Helen Kleinfelder^1^, Sudheer Madan Mohan Gambheer^1^, Cornelia Endres^1^, Sabina Schaberg^1^, Dominik Schmidt^1^, Gerin Prince^2^, Irene Gonzalez-Menendez^4^, Detlef Bentrop^5^, Rainer Trittler^6^, Svetlana Rylova^7^, Dietmar Pfeifer^1^, Geoffroy Andrieux^8,9^, Leticia Quintanilla-Martinez^4^, Anna Lena Illert^1,8^, Nikolas von Bubnoff^2^, Robert Zeiser^1^, and Justus Duyster*^1,8^

**Supplementary Figure 1**

**
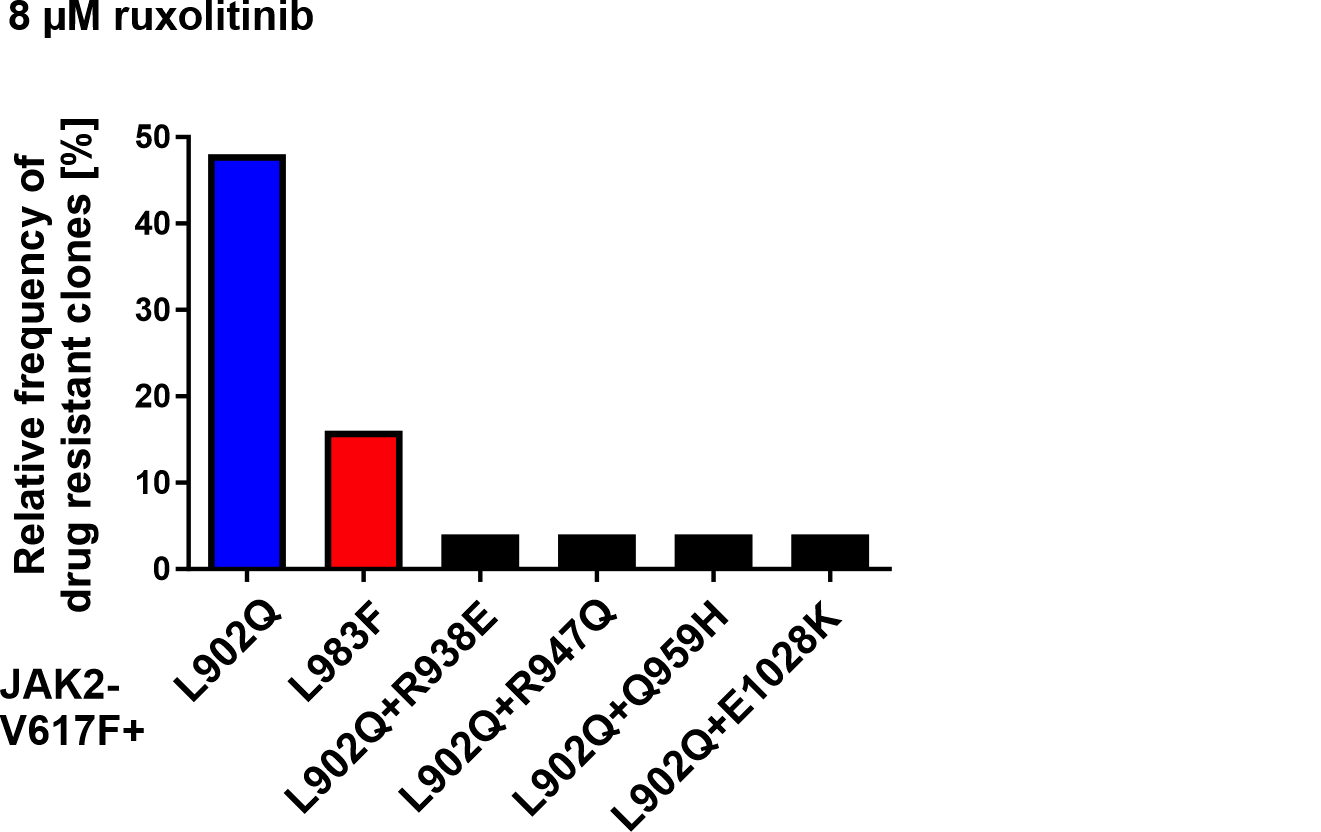
**

**Supplementary Fig. 1**. **Frequency of drug resistant Ba/F3-JAK2-V617F clones in a mutagenesis screen using 8 µM ruxolitinib**. Ba/F3 cells transformed by JAK2-V617F were pretreated with the chemical mutagen *N*-ethyl-*N*-nitrosourea twice for 12 h at a concentration of 50 μg/ml before performing the screening. Resistant clones from the screen were sequenced and analyzed for JAK2 mutations. N=1.

**Supplementary Figure 2**


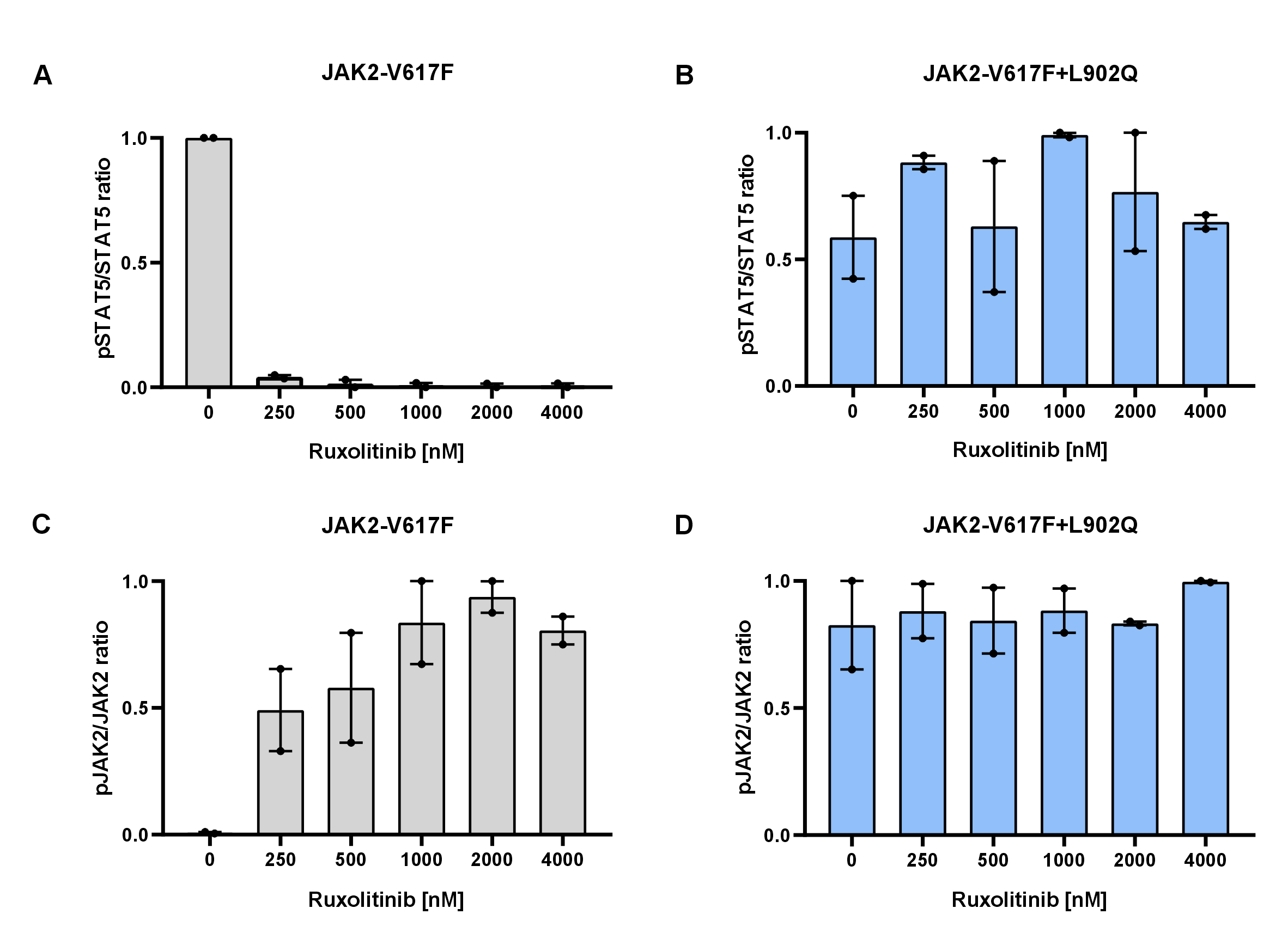


**Supplementary Fig. 2**. **JAK2-V617F+L902Q^+^ cells are ruxolitinib resistant and display persistent STAT5 activation**. **(A)** Quantitative ratio of pSTAT5 versus STAT5 of JAK2-V617F expressing Ba/F3 cells in presence of indicated concentrations of ruxolitinib. N=2 independent experiments. **(B)** Quantitative ratio of pSTAT5 versus STAT5 of JAK2-V617F+L902Q expressing Ba/F3 cells in presence of indicated concentrations of ruxolitinib. N=2 independent experiments. **(C)** Quantitative ratio of pJAK2 versus total JAK2 of JAK2-V617F expressing Ba/F3 cells immunoblot in presence of indicated concentrations of ruxolitinib. N=2 independent experiments. **(D)** Quantitative ratio of pJAK2 versus total JAK2 of JAK2- V617F+L902Q expressing Ba/F3 cells in presence of indicated concentrations of ruxolitinib. N=2 independent experiments. Data represent mean ± SD.

**Supplementary Figure 3**


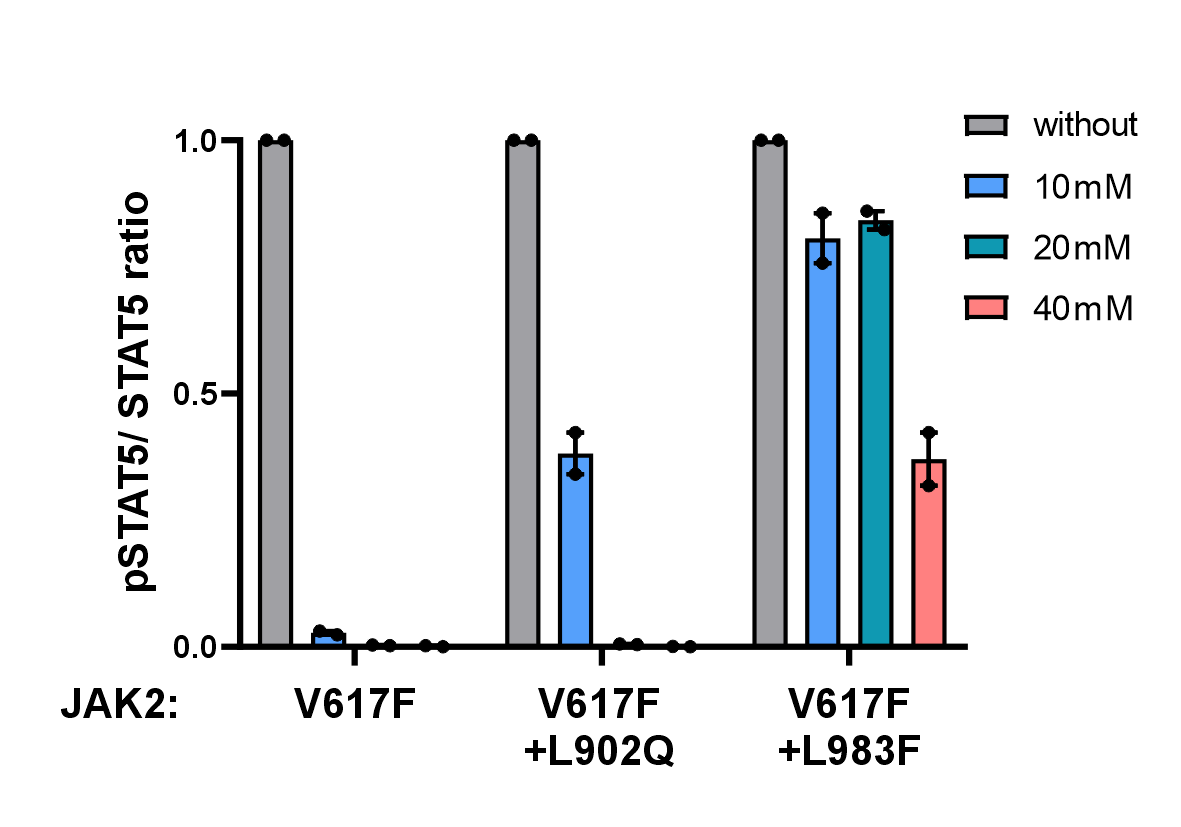


**Supplementary Fig. 3**. **JAK2-V617F+L98F mediates ruxolitinib resistance.** Quantitative ratio of pSTAT5 versus total STAT5 of JAK2-V617F, JAK2-V617F+L902Q and JAK2-V617F+L983F expressing Ba/F3 cells in presence of indicated concentrations of ruxolitinib. (n=2) independent experiments. Data represent mean ± SD.

**Supplementary Figure 4**


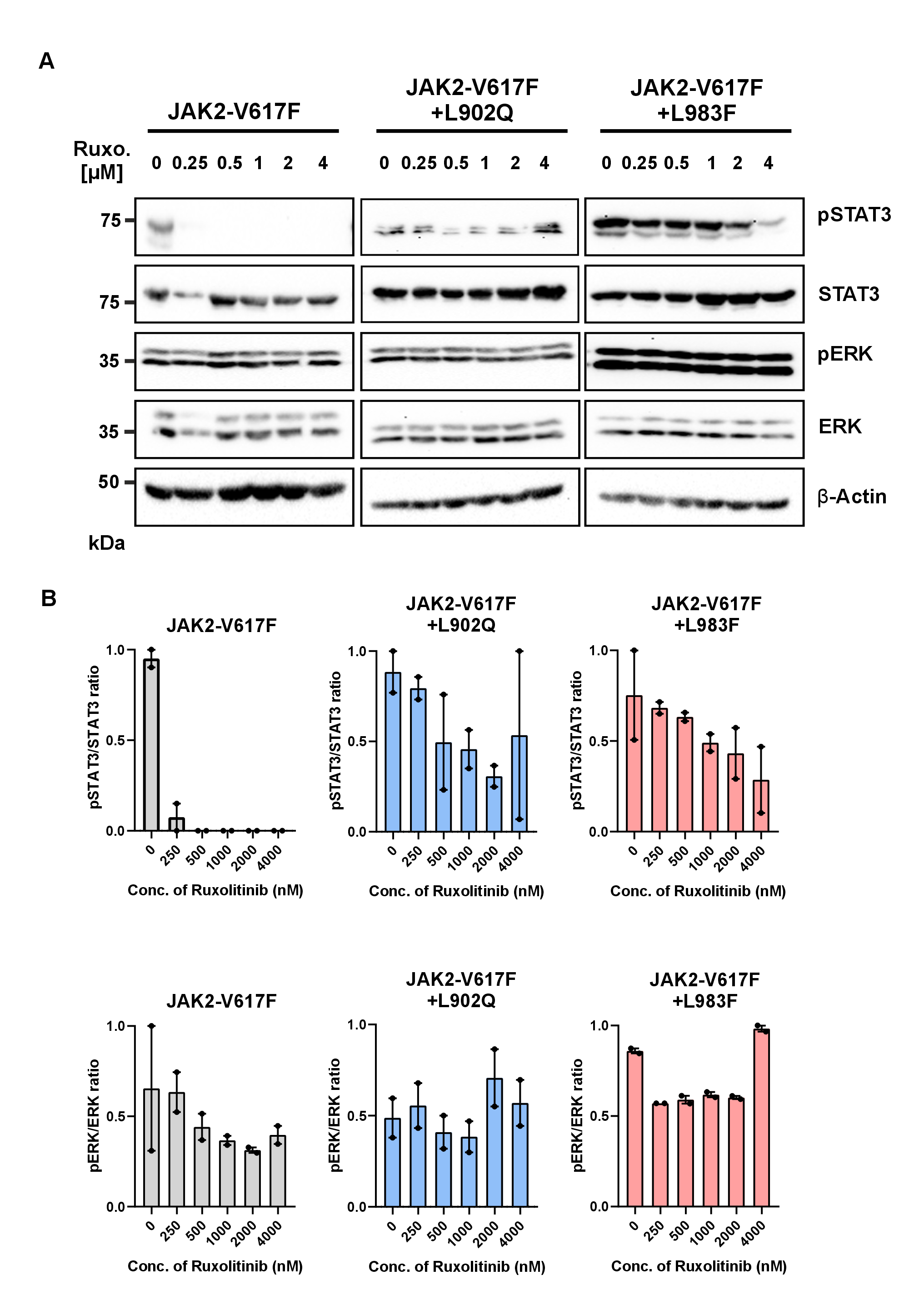


**Supplementary Fig. 4**. **JAK2-V617F+L902Q and JAK2-V617F+L98F positive cells display persistent activation of STAT3, and ERK upon ruxolitinib. (A)** Immunoblot analysis of JAK2-V617F, JAK2-V617F+L902Q and JAK2-V617F+L983F expressing Ba/F3 cells in presence of indicated concentrations of ruxolitinib. A representative image of n=2 two independent experiments is shown. For JAK2-V617F and JAK2-V617F+L983, the samples derive from the same experiment but different gels for pSTAT3, total STAT3 and β-actin, another for pERK and total ERK were processed in parallel. For JAK2-V617F and JAK2-V617F+L902Q, the samples derive from the same experiment but different gels for pSTAT3, total STAT3 and β-actin, another for pERK and total ERK were processed in parallel. Here, only the blot of JAK2-V617F+L902Q is shown. Uncropped images are provided as a source data file. **(B)** Quantitative ratio of pSTAT3 versus total STAT3, and pERK1/2 versus total ERK1/2 of JAK2-V617F, JAK2-V617F+L902Q, and JAK2-V617F+L983F expressing Ba/F3 cells in presence of indicated concentrations of ruxolitinib. Data represent mean ± SD.

**Supplementary Figure 5**


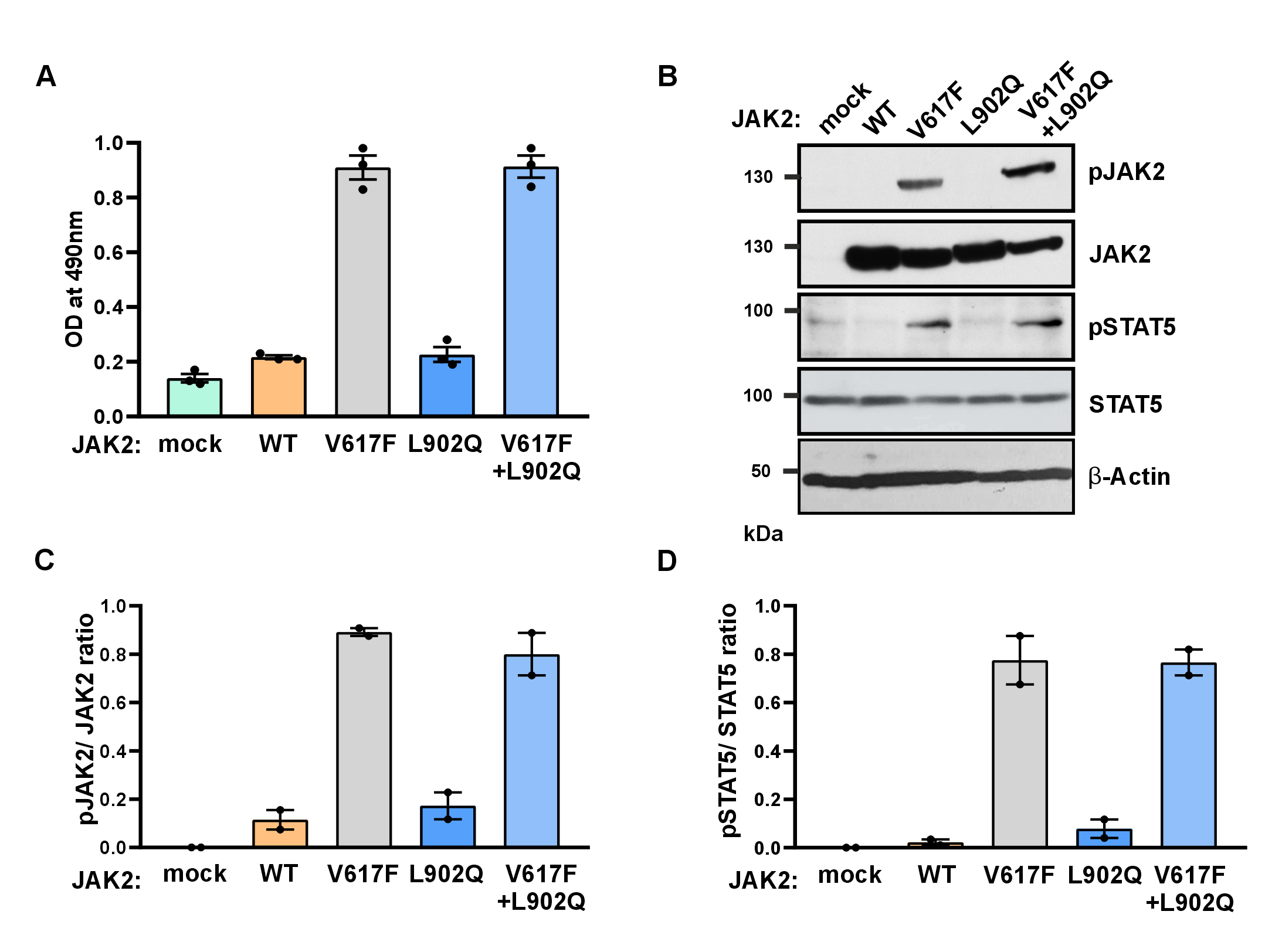


**Supplementary Fig.5. JAK2-V617F+L902Q shows transformation ability similar to JAK2-V617F *in vitro.* (A)** Ba/F3 cells stably expressing JAK2 mutants were cultivated in absence of IL-3 for four days. Cell growth determined by 490 nm optical density (OD). Data represent mean ± SD. ***p< 0.001, n.s., not significant, p>0.05 by Student’s t test. Technical replicates (n=3) shown from one representative experiment of n=2 independent experiments. **(B)** Immunoblot analysis of Ba/F3 cells showing similar STAT5 activation for JAK2-V617F and JAK2-V617F+L902Q expressing cells. A representative image of n=2 two independent experiments is shown. Data represent mean ± SD. The samples derive from the same experiment but different gels for pJAK2, total JAK2 and β-actin, another for pSTAT5 and total STAT5 were processed in parallel. Uncropped images are provided as a source data file.  **(C)** Quantitative ratio of pJAK2 versus total JAK2 of Ba/F3 cells. Data represent mean ± SD. **(D)** Quantitative ratio of pSTAT5 versus total STAT5 of Ba/F3 cells. Data represent mean ± SD.

**Supplementary Figure 6**


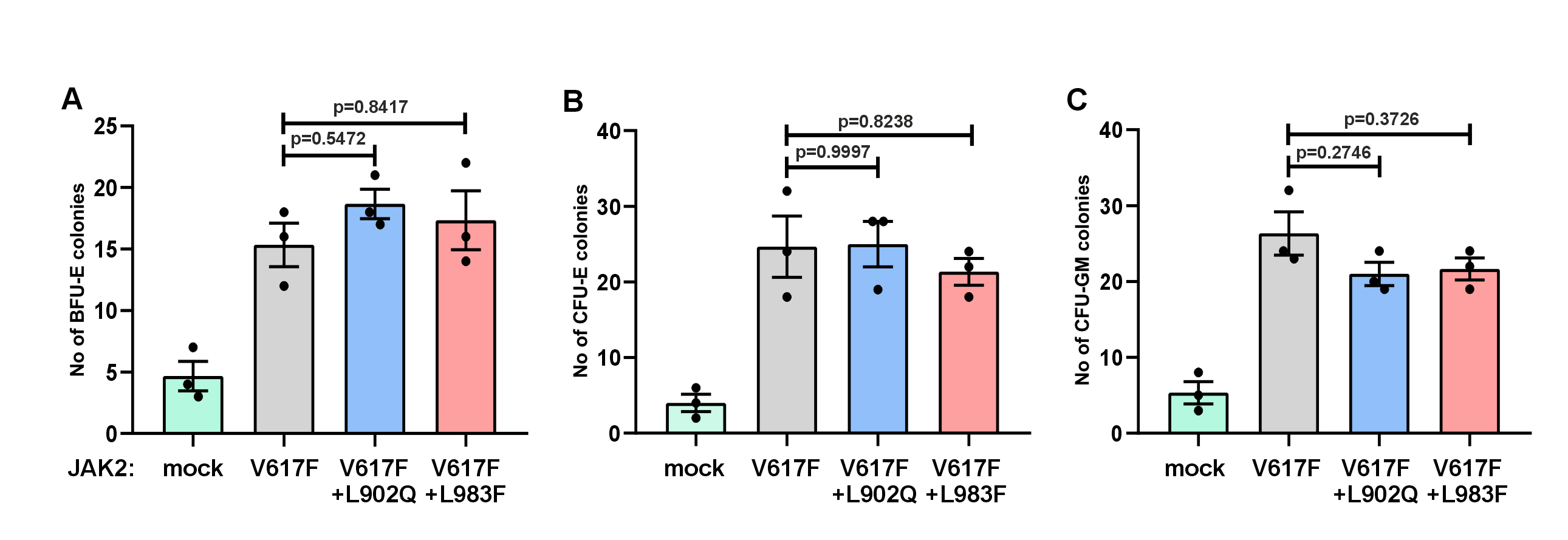


**Supplementary Fig. 6**. **JAK2-V617F+L902Q and JAK2-V617F+L98F positive cells display numbers of colony forming units (CFU) similar to JAK2-V617F positive cells. (A)** Burst forming units (BFU-E), **(B)** CFU-E or **(C)** CFU-GM of primary bone marrow cells from Balb/C mice transduced with empty vector (mock), JAK2-V617F, JAK2-V617F+L902Q, or JAK2-V617F+L983F were sorted for EGFP^+^ cells. 2000 EGFP^+^ cells were plated in methyl cellulose medium in absence of cytokines. Colony forming units were calculated after two weeks. Burst forming units (BFU-E) were calculated after seven days. Data represent mean ± SD. P-value was calculated using one-way ANOVA test. N=3 (cells transduced from different Balb/C mice).

**Supplementary Figure 7**


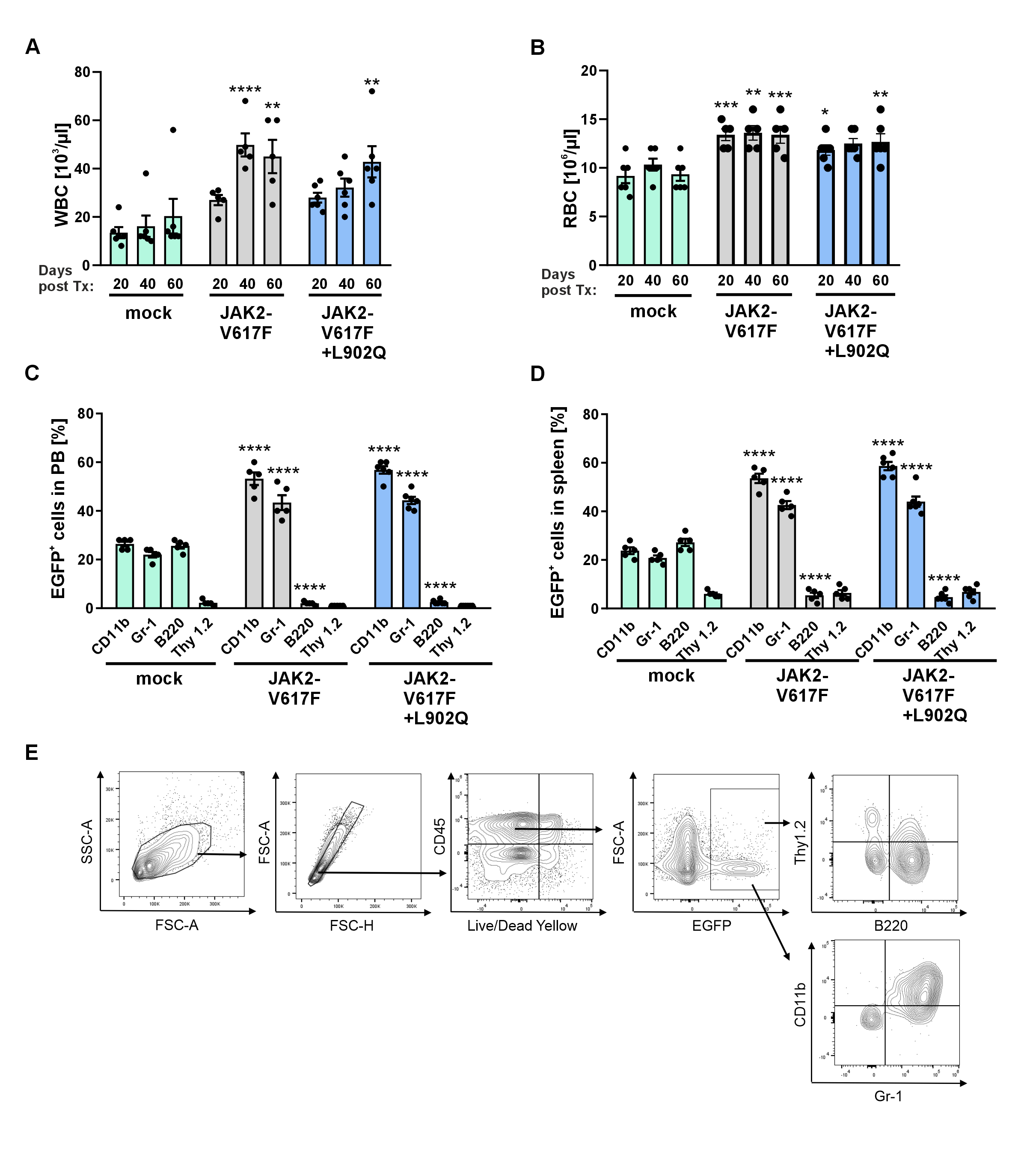


**Supplementary Fig. 7**. **JAK2-V617F+L902Q mice develop an MPN similar to JAK2-V617F animals. (A)** White blood counts (WBC) from mice transplanted with empty vector (mock) (n=5), JAK2-V617F (n=5), or JAK2-V617F+L902Q (n=6) at 20, 40, or 60 days after transplantation. Data represent mean ± SEM. Statistical significance was determined using two-way ANOVA test. (p=0.1408 mock vs JAK2-V617F; p=0.0859 mock vs JAK2-V617F+L902Q at day 20; ****p< 0.0001 mock vs JAK2-V617F mice at day 40. p=0.0524 mock vs JAK2-V617F+L902Q mice at 40; **p=0.0028 mock vs JAK2-V617F at day 60; p=0.0043 mock vs JAK2-V617F+L902Q mice at 60). **(B)** red blood counts (RBC) from mice transplanted with empty vector (mock) (n=5), JAK2-V617F (n=5), or JAK2-V617F+L902Q (n=6) at 20, 40, or 60 days after transplantation. Data represent mean ± SEM. Statistical significance was determined using two-way ANOVA test. Data represent mean ± SEM. Statistical significance was determined using two-way ANOVA test. ((***p= 0.0003 mock vs JAK2-V617F; p=0.0196 mock vs JAK2-V617F+L902Q at day 20; **p<=0.0056 mock vs JAK2-V617F mice at day 40. p=0.0682 mock vs JAK2-V617F+L902Q mice at 40; ***p=0.0005 mock vs JAK2-V617F at day 60; **p=0.0029 mock vs JAK2-V617F+L902Q mice at 60). **(C+D)** Flow cytometric analyses of EGFP^+^ cells in **(C)** peripheral blood (PB) at day 60 in JAK2-V617F and JAK2-V617F+L902Q animals as compared to mock animals. Data represent mean ± SEM. ****p< 0.0001 mock CD11b vs JAK2-V617F and ****p< 0.0001 mock CD11b vs JAK2-V617F+L902Q; ****p< 0.0001 mock Gr-1 vs JAK2-V617F and ****p< 0.0001 mock Gr-1 vs JAK2-V617F+L902Q; ****p< 0.0001 mock B220 vs JAK2-V617F and ****p< 0.0001 mock B220 vs JAK2-V617F+L902Q. n.s: non-significant p=0.8228 in mock Thy1.2 versus JAK2-V617F and p=0.8084 mock Thy1.2 vs JAK2-V617F+L902Q; **(D)** splenocytes at day 90 after transplant reveal similar increases of myeloid cells (CD11b^+^, Gr-1^+^) in JAK2-V617F and JAK2-V617F+L902Q animals as compared to mock animals. Data represent mean ± SEM. Statistical significance was determined using two-way ANOVA test. ****p< 0.0001 mock CD11b vs JAK2-V617F and ****p< 0.0001 mock CD11b vs JAK2-V617F+L902Q; ****p< 0.0001 mock Gr-1 vs JAK2-V617F and ****p< 0.0001 mock Gr-1 vs JAK2-V617F+L902Q; ****p< 0.0001 mock B220 vs JAK2-V617F and ****p< 0.0001 mock B220 vs JAK2-V617F+L902Q. n.s: non-significant p=0.9798 in mock Thy1.2 vs JAK2-V617F and p=0.9082 in mock Thy1.2 vs JAK2-V617F+L902Q. **(E)** Gating strategy applied to data in (C) and (D) or main Figure 2E.

**Supplementary Figure 8**


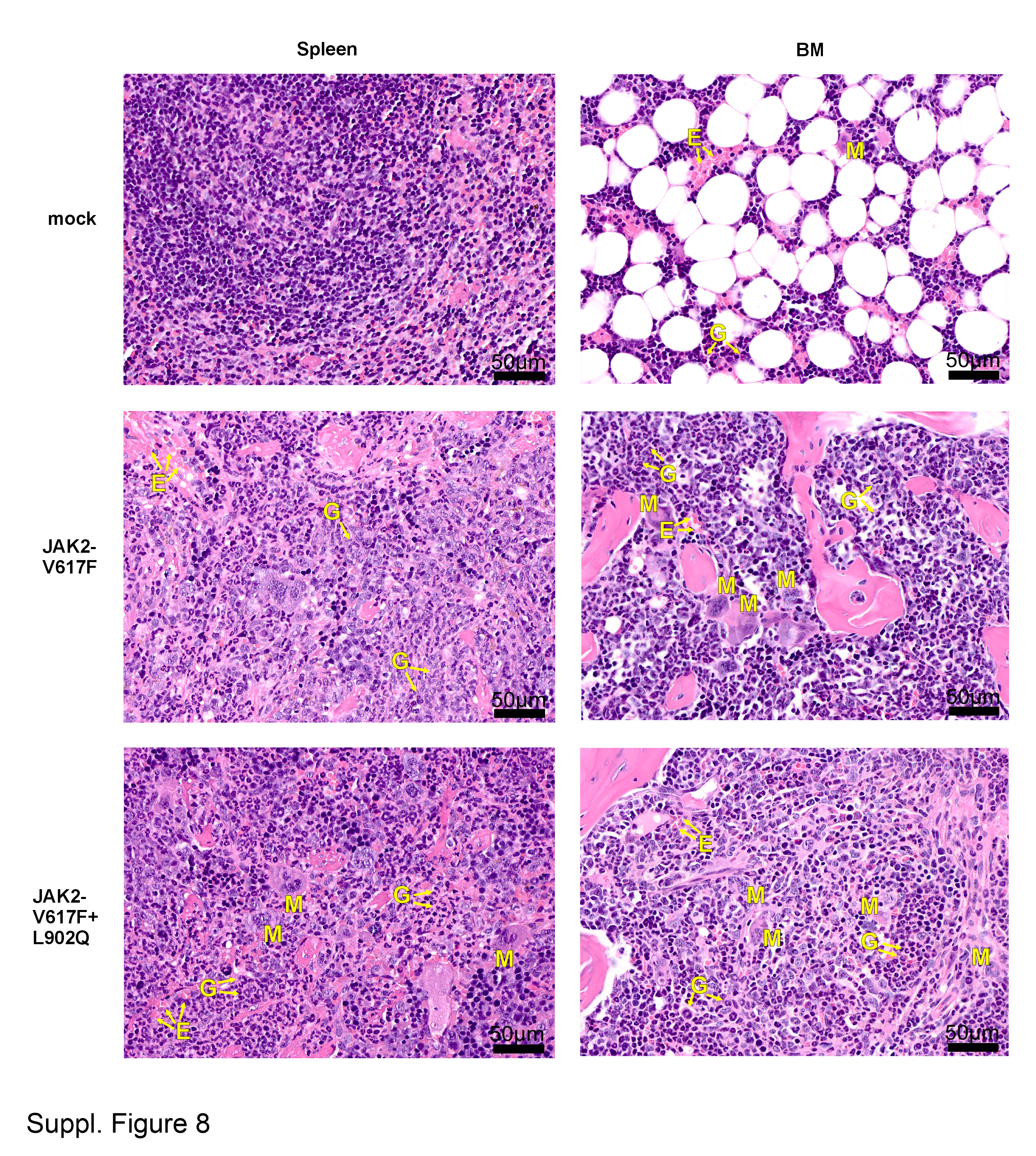


**Supplementary Fig. 8**. **JAK2-V617F+L902Q mice display MPN phenotype and myelofibrosis similar to JAK2-V617F mice.** Enlarged version of Figure 2F. Histopathologic H&E stainings of liver, spleen and BM from JAK2-V617F and JAK2-V617F+L902Q mice reveal hyperplastic, left-shifted myelopoiesis granulopoiesis (G), erythropoiesis (E), and moderately increased megakaryopoiesis (M) in bone marrow. Gomori reticular fiber staining shows marked myelofibrosis. Note the extramedullary hematopoiesis in liver and spleen secondary to BM myelofibrosis. In contrast the mock animal shows normal liver, spleen and BM. (all figures 400x)

**Supplementary Figure 9**


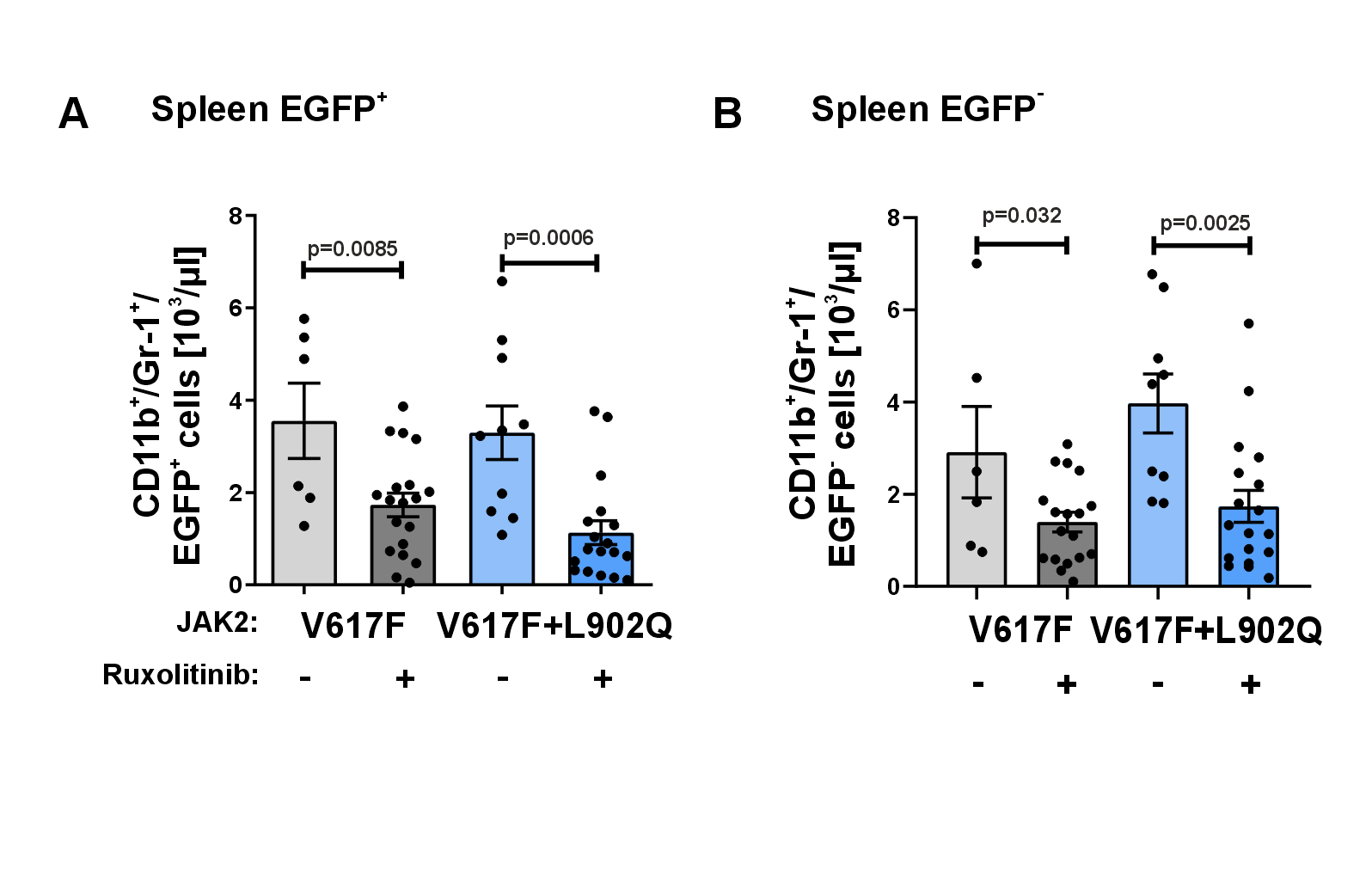


**Supplementary Fig. 9: Ruxolitinib treatment reduces myeloid cells of malignant (EGFP^+^) and non-malignant (EGFP^-^) origin. (A+B)** Flow cytometric analysis of splenocytes at day 90 after transplant reveals similar effects of ruxolitinib treatment on **(A)** EGFP^+^ and **(B)** EGFP^-^ CD11b^+^/Gr-1^+^ cells in JAK2-V617F and JAK2-V617F+L902Q animals (n=18/15, respectively) compared to vehicle treated group (n=6/7). Data represent mean ± SEM. Statistical significance was determined using two-tailed Student’s t test.

**Supplementary Figure 10**


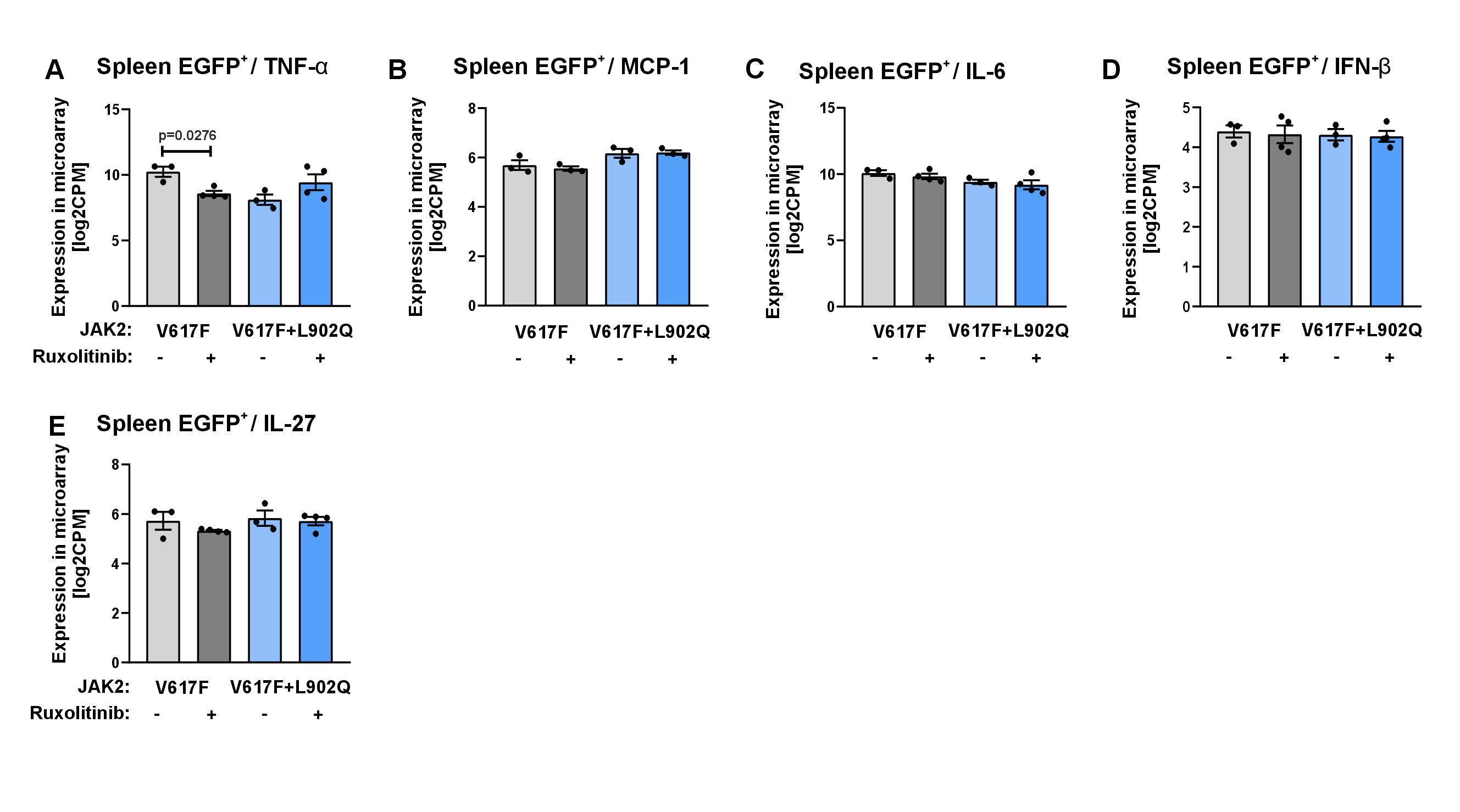


**Supplementary Fig. 10: Ruxolitinib mainly does not affect inflammatory cytokines levels from cells of malignant (EGFP^+^) origin.** EGFP^+^**/**Gr-1^+^/CD11b^+^ granulocytes from the spleen of vehicle and ruxolitinib treated JAK2-V617F (n=3 vs. 4) and JAK2-V617F+L902Q (n=3 vs 4) mice were isolated. Microarray analysis was performed. Inflammatory cytokines tumor necrosis factor alpha (TNF-α), MCP-1/ CCL2, IL-6, IFN-β, and IL-27 are depicted. Data represent mean ± SD. Statistical significance was determined using two-tailed Student’s t test.

**Supplementary Figure 11**


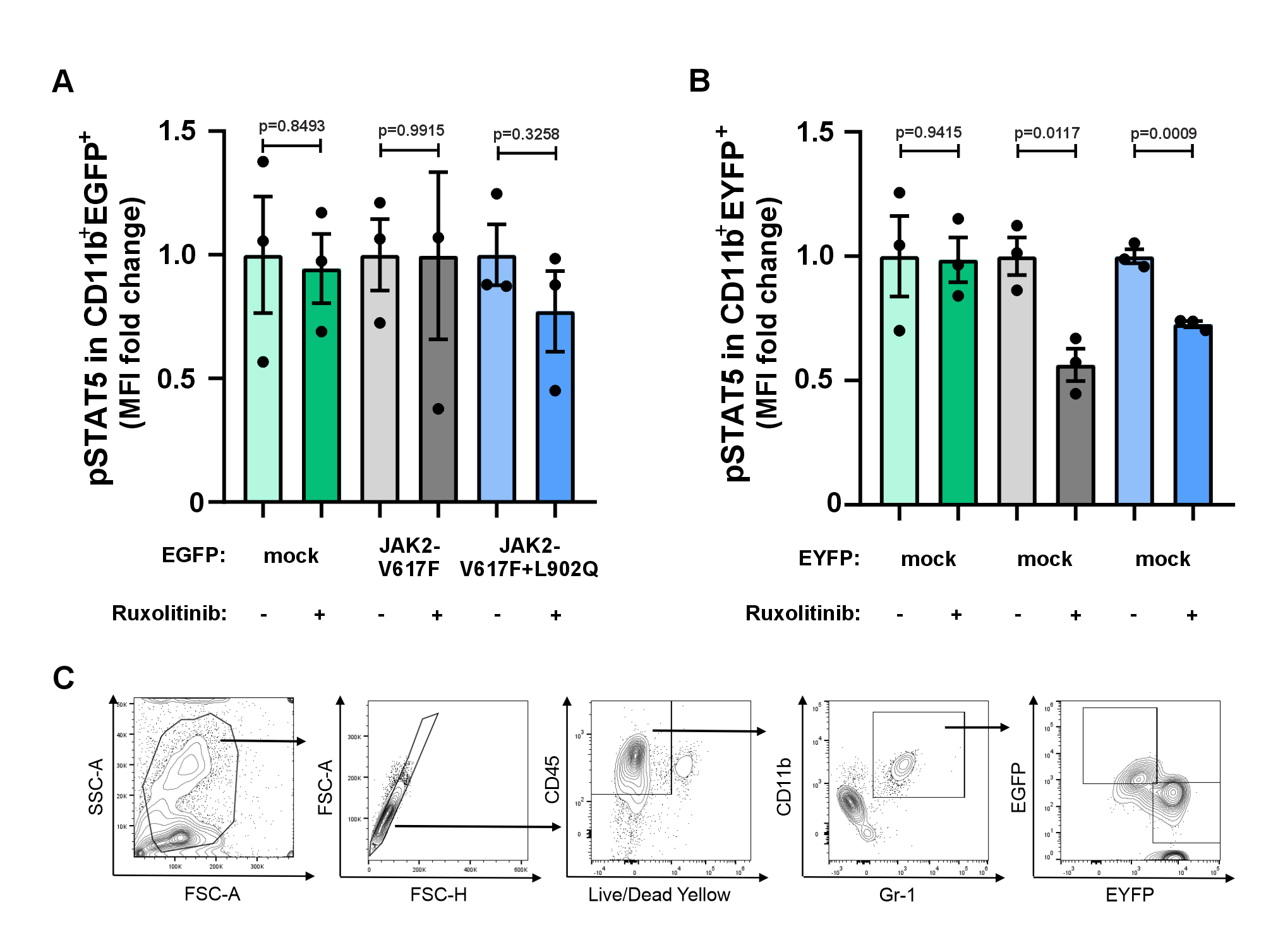


**Supplementary Fig. 11: Ruxolitinib treatment reduces STAT5 phosphorylation in myeloid cells of non-malignant (EGFP^-^) origin. (A)** Intracellular flow cytometric analysis of at day 40 after transplant for peripheral blood Gr-1^+^/CD11b^+^ **(A)** EGFP^+^ and **(B)** EYFP^+^ cells from mock, JAK2-V617F, or JAK2-V617F+L902Q animals, treated with vehicle or ruxolitinib (n=4-5, respectively). Data represent mean ± SEM. Statistical significance was determined using unpaired Student’s t test. **(C)** Gating strategy applied to data in (A) and (B).

**Supplementary Figure 12**


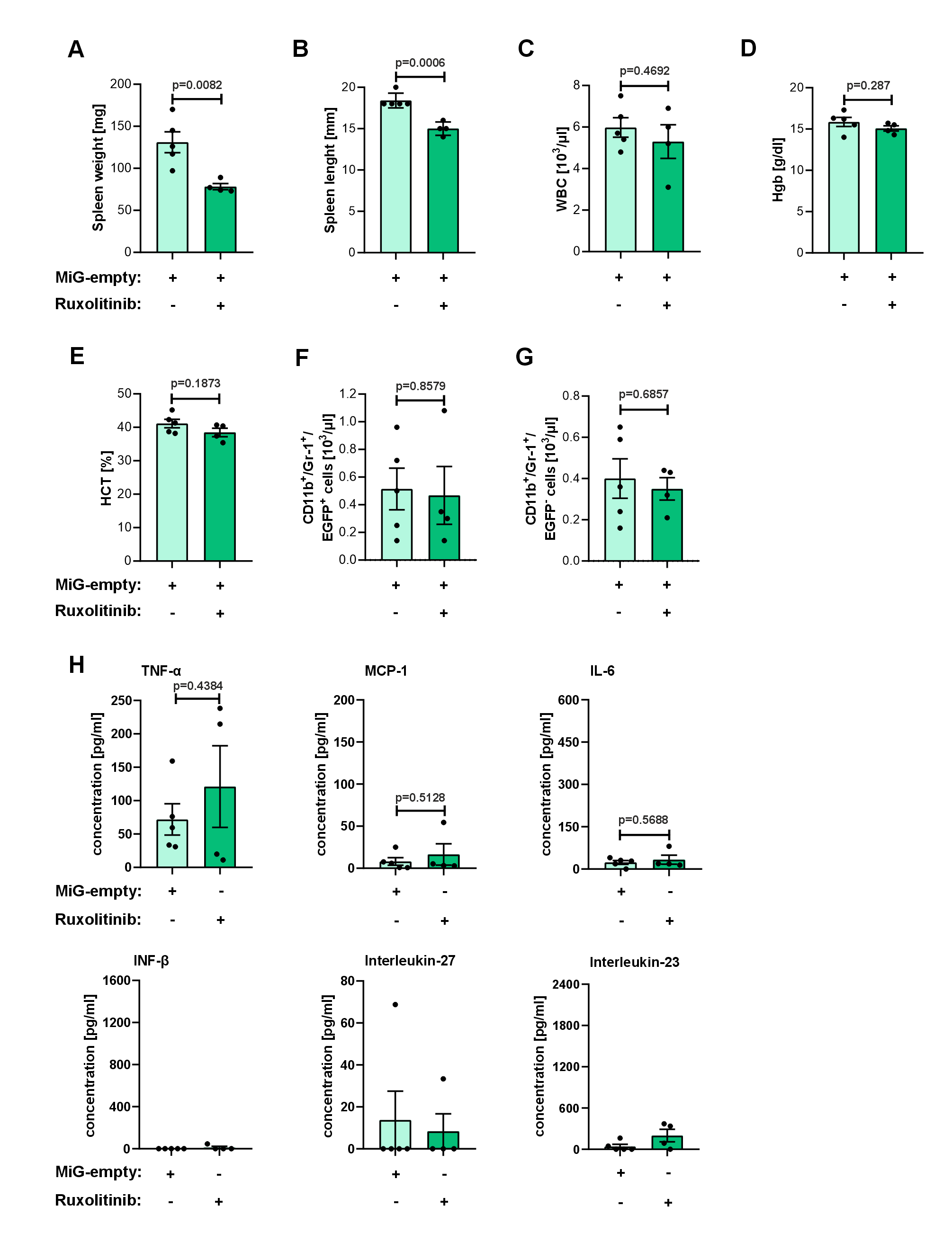


**Supplementary Fig. 12**. **Ruxolitinib treated control mice display reduced spleen size but no changes in peripheral blood values or cytokines levels. (A-B)** MiG-empty control mice treated with ruxolitinib display reduced spleen **(A)** weight and **(B)** length compared to vehicle treated mice. N=4/5. **(C-E)** Ruxolitinib-treated control mice do not exhibit changes in **(C)** white blood cell counts (WBC), **(D)** hemoglobin (HGB), or **(E)** hematocrit (HCT). **(F-G)** Flow cytometric analysis of PB cells at day 64 showing no differences in **(F)** CD11b^+^/Gr-1^+^/EGFP^+^ or **(G)** -EGFP^-^ cells. Data represent mean ± SEM. Statistical significance was determined using unpaired Student’s t test.

**Supplementary Figure 13**


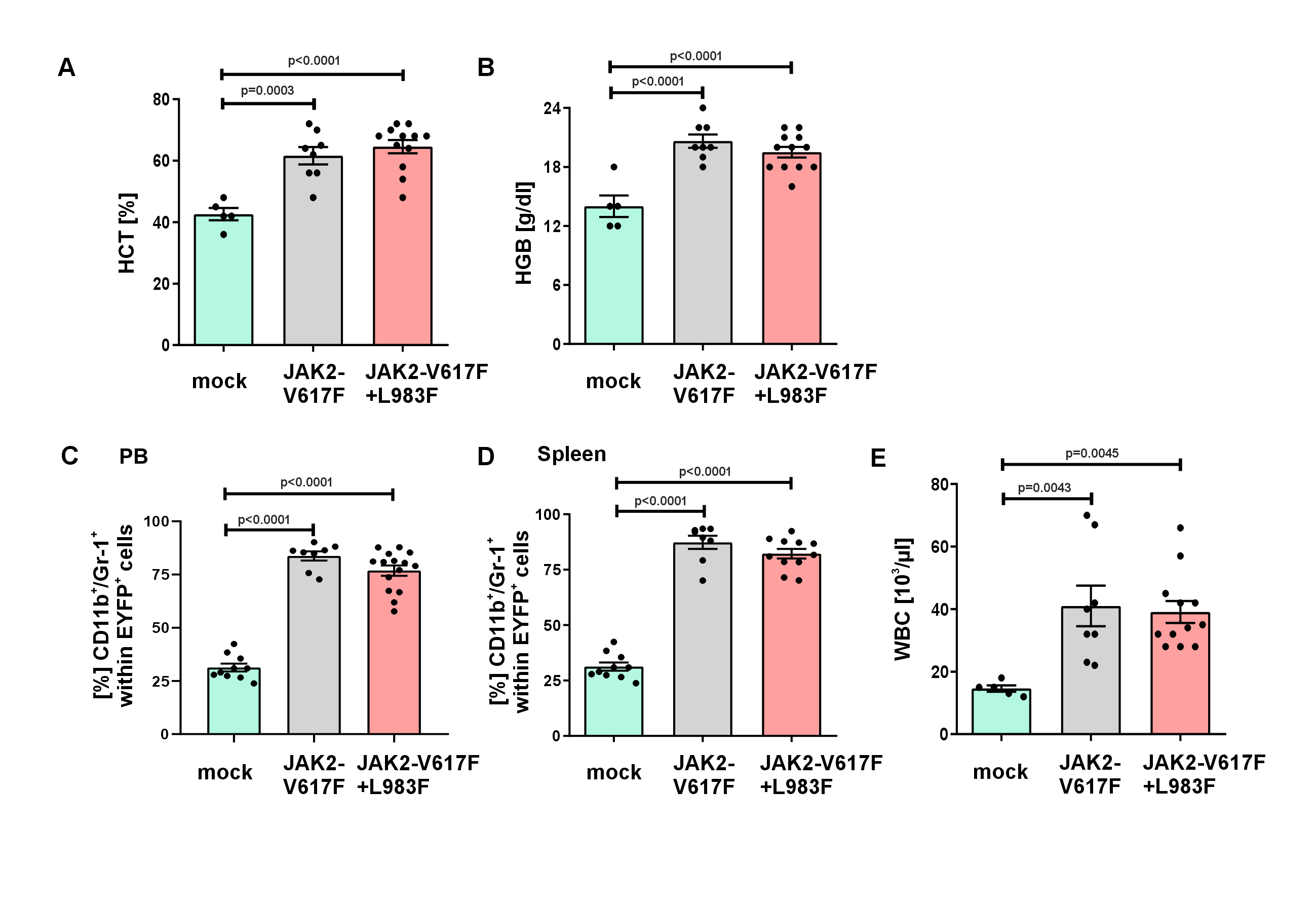


**Supplementary Fig. 13. JAK2-V617F+L983F mice develop an MPN similar to JAK2-V617F animals. (A, B)** Similar increase of **(A)** hematocrit (HCT), and **(B)** hemoglobin (HGB) of JAK2-V617F (n=5) and JAK2-V617F+L983F (n=8) compared to empty vector (mock; n=5) mice at day 20 after transplantation. **(C)** EYFP^+^Gr-1^+^/CD11b^+^ PB cells, **(D)** EYFP^+^Gr-1^+^/CD11b^+^ splenocytes, and **(E)** white blood cell count (WBC) of JAK2-V617F, JAK2-V617F+L983F, and empty vector mice at day 40 after transplantation. Data represent mean ± SEM. **p< 0.01, ***p< 0.001. Statistical significance was determined using one-way ANOVA test.

**Supplementary Figure 14**


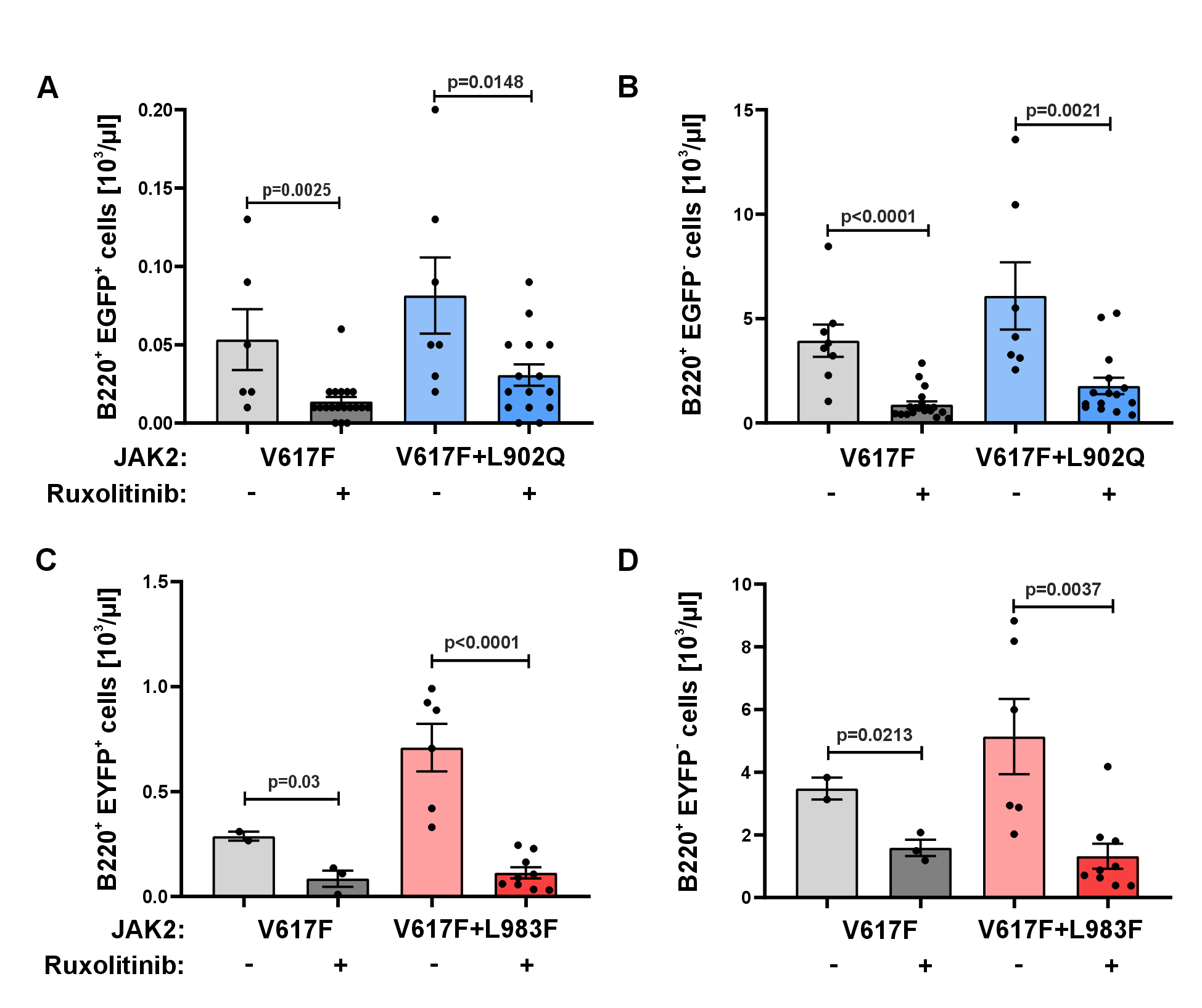


**Supplementary Fig. 14: Ruxolitinib treatment reduces B cell numbers of malignant (EGFP^+^) and non-malignant (EGFP^-^) origin. (A-B)** Flow cytometric analysis at day 90 after transplantion reveals similar effects of ruxolitinib treatment on **(A)** EGFP^+^ and **(B)** EGFP^-^ B220^+^ cells in JAK2-V617F or JAK2-V617F+L902Q animals (n=19/15, respectively). **(C-D)** Flow cytometric analysis at day 70 after transplant reveals similar effects of ruxolitinib treatment on **(C)** EYFP^+^ and **(D)** EYFP^-^ B220^+^ cells in JAK2-V617F or JAK2-V617F+L983F animals (n=7/8, respectively). Data represent mean ± SD. *p< 0.05, **p< 0.01. Statistical significance was determined using two-tailed Student’s t test.

**Supplementary Figure 15**

**
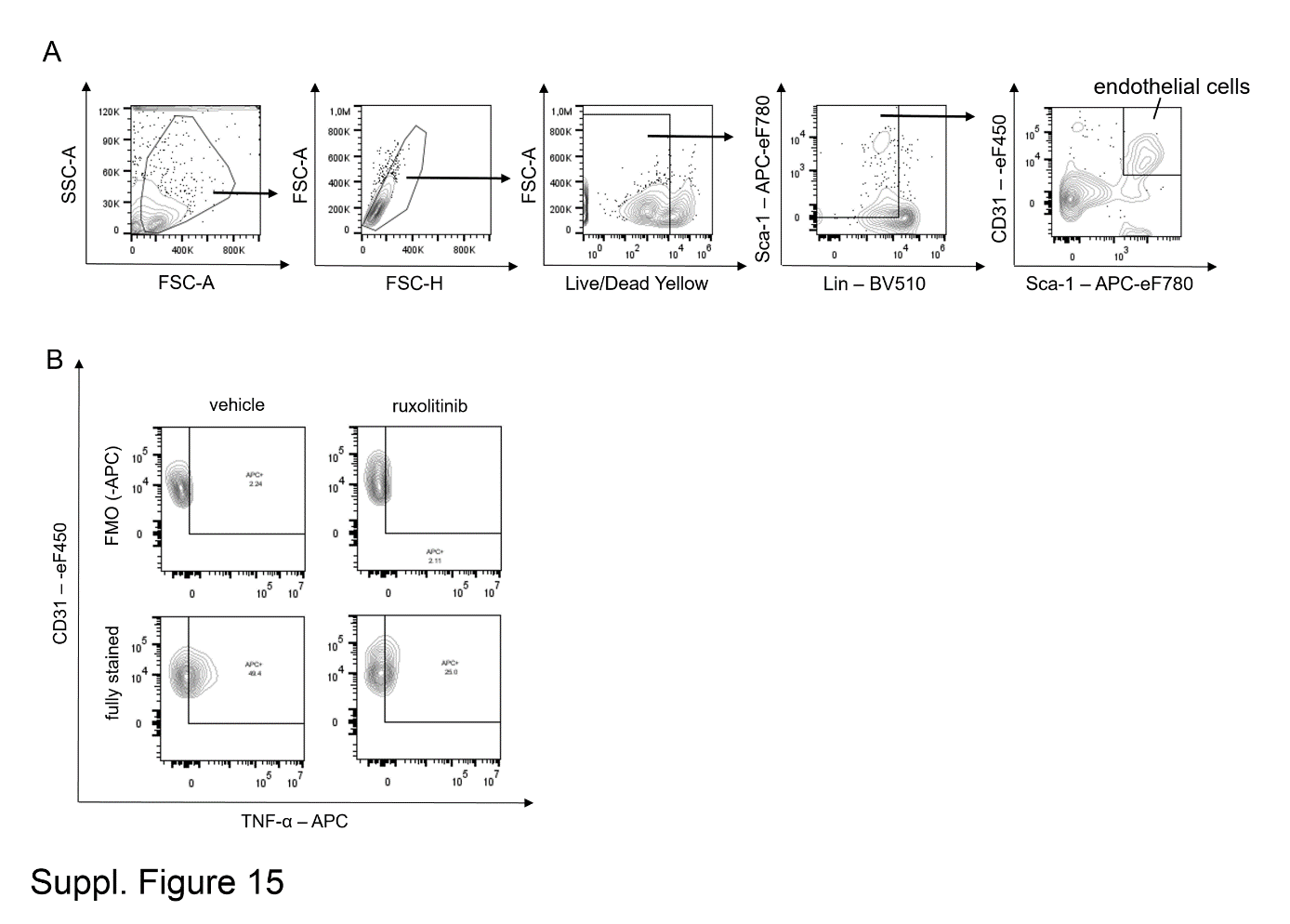
**

**Supplementary Fig. 15: Ruxolitinib treatment reduces inflammatory cytokine levels of endothelial cells. (A-B)** Gating strategy for FACS analysis of bone marrow stromal endothelial cells (main Figure 6D-F). Sca-1^+^CD31^+^ cells were stained for intracellular cytokines, as for instance **(B)** TNF-α.

**Supplementary Figure 16**


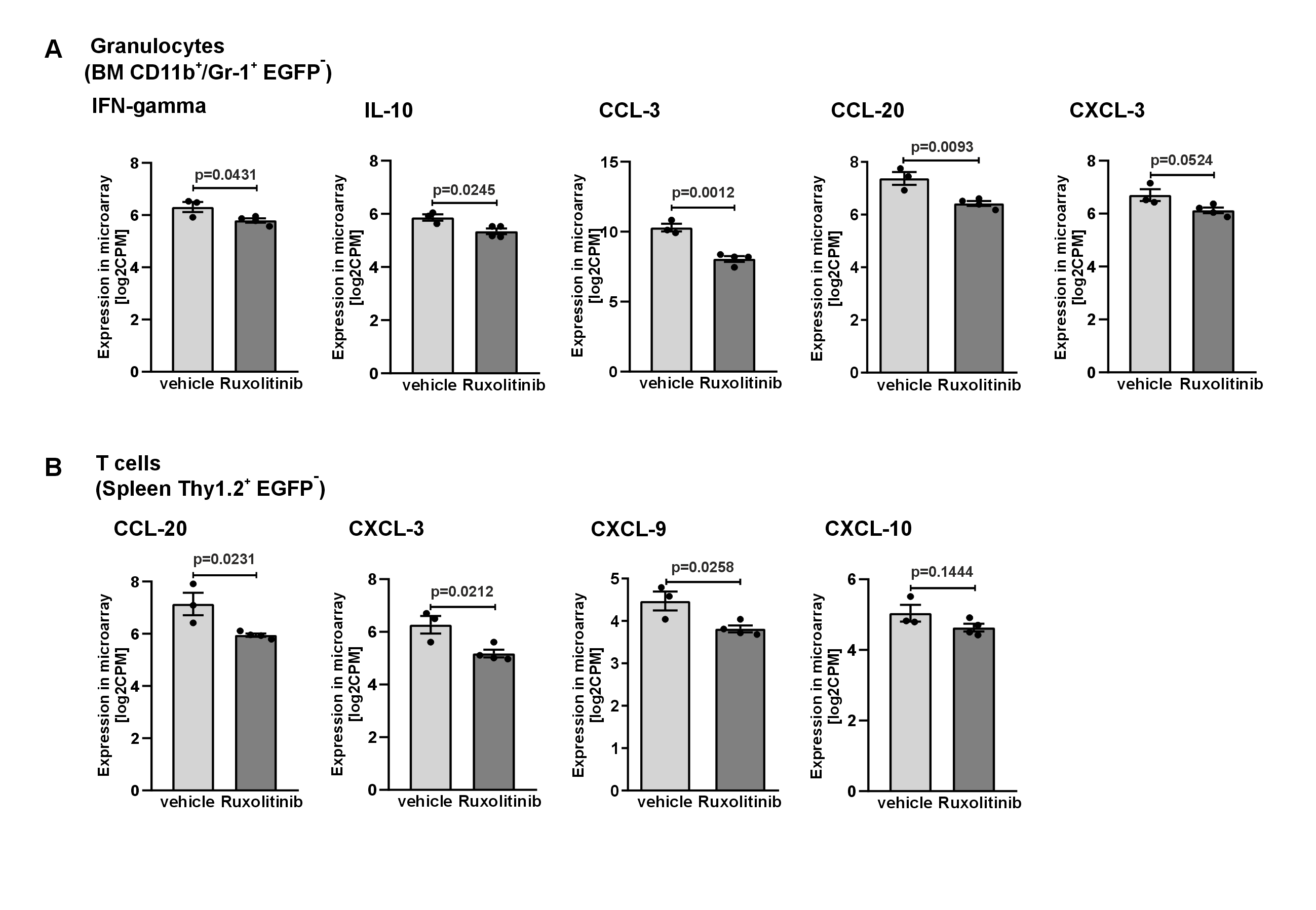


**Supplementary Fig. 16: Ruxolitinib downregulates the transcript levels of inflammatory cytokines and chemokines in cells of non-malignant origin. (A)** Bone marrow EGFP^-^ Gr-1^+^/CD11b^+^ granulocytes and spleen EGFP^-^ Thy1.2^+^ T cells were isolated from vehicle (n=3) and ruxolitinib (n=3) treated JAK2-V617F mice. Cytokines INF-gamma and IL-10, and chemokines CCL-3, CCL-20, CXCL-3, and CXCL-9 levels are depicted. **(B)** Spleen EGFP^-^ Thy 1.2^+^ were isolated from vehicle (n=3) and ruxolitinib (n=3) treated JAK2-V617F mice. Levels for several chemokines are depicted. Data represent mean ± SEM. *p< 0.05, **p< 0.01, ***p< 0.001, ****p< 0.0001. Statistical significance was determined using two-tailed Student’s t test.

**Supplementary Figure 17**


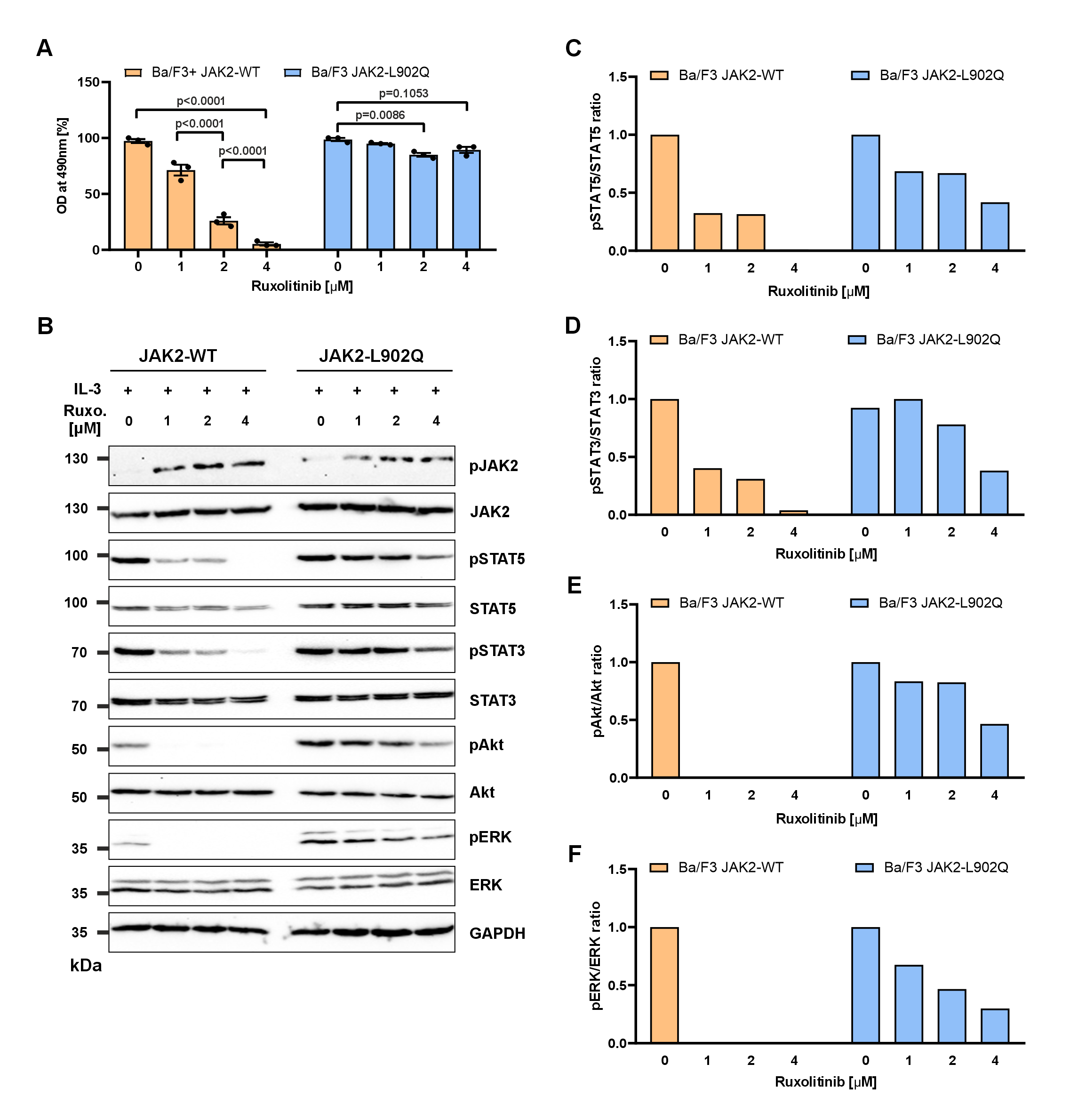


**Supplementary Fig. 17: JAK2-L902Q positive Ba/F3 cells display ruxolitinib resistance. (A)** MTT incorporation assay of JAK2-WT and JAK2-L902Q expressing Ba/F3 cells in presence of IL-3 with indicated ruxolitinib concentrations. Data represent mean ± SD. Statistical significance was determined using one-way ANOVA test. N=3 independent experiments. **(B)** Immunoblot analysis (n=1) of JAK2-WT, or JAK2-L902Q expressing Ba/F3 cells with indicated concentrations of ruxolitinib and IL-3. The samples derive from the same experiment but different gels for pJAK2, total JAK2, pAkt, total Akt, another for pSTAT3, total STAT3, another for pSTAT5, total STAT5, pERK, total ERK and GAPDH were processed in parallel. Uncropped images are provided as a source data file. **(C-F)** Quantitative ratio of pSTAT5 versus total STAT5, pSTAT3 versus total STAT3, pAKT versus total AKT, and pERK1/2 versus total ERK1/2 of JAK2-wild type and JAK2-L902Q expressing Ba/F3 cells with indicated concentrations of ruxolitinib and IL-3.

**Supplementary Figure 18**


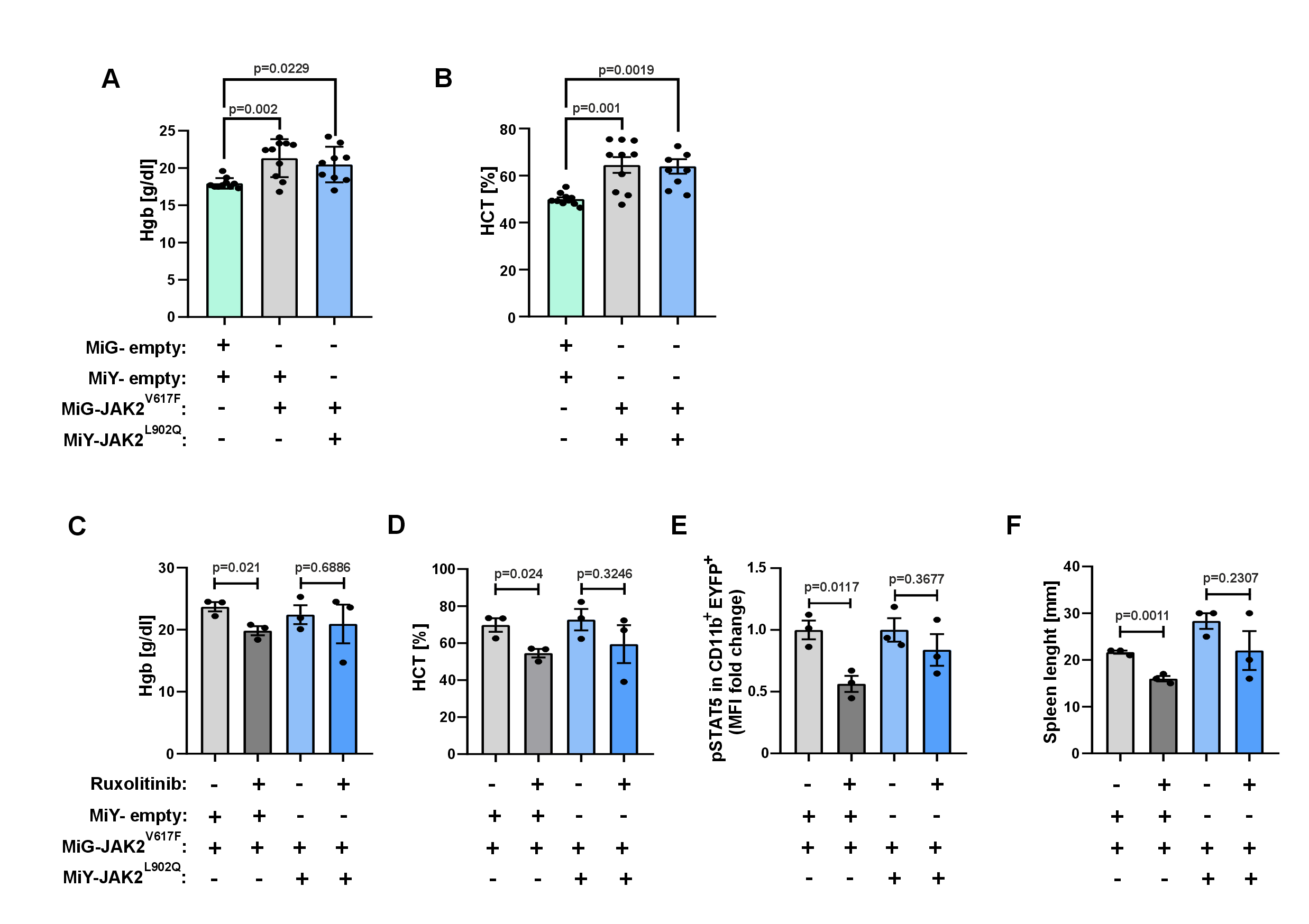


**Supplementary Fig. 18: Ruxolitinib shows reduced response in MiG-JAK2-V617F/MiY-JAK2-L902Q mice.** **(A-B)** JAK2-V617F/JAK2-L902Q chimera mice harboring ruxolitinib resistance in non-malignant bone marrow cells (MiG-JAK2-V617F/MiY-JAK2-L902Q) develop an MPN, charactericed by increased **(A)** Hgb, or **(B)** HCT. Data represent mean ± SD. Statistical significance was determined using one-way ANOVA test. **(C-D)** Ruxolitinib treatment did not significantly decrease **(D)** Hgb, or **(E)** HCT in JAK2-V617F/JAK2- L902Q chimera mice. Data represent mean ± SEM. **(E**) Intracellular FACS staining of STAT5 phosphorylation was not significantly decreased in JAK2-V617F/JAK2- L902Q chimera mice in contrast to JAK2-V617F/empty mice. Data represent mean ± SEM. **(F)** No significant spleen size reduction in JAK2-V617F/JAK2- L902Q chimera mice. N≥3 (each data point indicates one specimen=mouse). Data represent mean ± SEM. Statistical significance was determined using two-tailed Student’s t test.
