# Supplementary material for: Efficacy of JAK1/2 inhibition in murine myeloproliferative neoplasms is not mediated by targeting oncogenic signaling
Source: Nat Commun. 2025 May 24;16:4833. doi: 10.1038/s41467-025-60019-6 (PMC12103521; doi:10.1038/s41467-025-60019-6)
Supplement: Supplementary file 2 — Reporting Summary [file 41467_2025_60019_MOESM2_ESM.pdf]

nature portfolio

Corresponding author(s): Justus Duyster

Last updated by author(s): Apr 17, 2025

Reporting Summary

Nature Portfolio wishes to improve the reproducibility of the work that we publish. This form provides structure for consistency and transparency in reporting. For further information on Nature Portfolio policies, see our Editorial Policies and the Editorial Policy Checklist.

Statistics

For all statistical analyses, confirm that the following items are present in the figure legend, table legend, main text, or Methods section.

n/a	Confirmed	
☐	☒	The exact sample size (*n*) for each experimental group/condition, given as a discrete number and unit of measurement
☐	☒	A statement on whether measurements were taken from distinct samples or whether the same sample was measured repeatedly
☐	☒	The statistical test(s) used AND whether they are one- or two-sided<br>*Only common tests should be described solely by name; describe more complex techniques in the Methods section.*
☒	☐	A description of all covariates tested
☒	☐	A description of any assumptions or corrections, such as tests of normality and adjustment for multiple comparisons
☐	☒	A full description of the statistical parameters including central tendency (e.g. means) or other basic estimates (e.g. regression coefficient) AND variation (e.g. standard deviation) or associated estimates of uncertainty (e.g. confidence intervals)
☐	☒	For null hypothesis testing, the test statistic (e.g. *F*, *t*, *r*) with confidence intervals, effect sizes, degrees of freedom and *P* value noted<br>*Give P values as exact values whenever suitable.*
☒	☐	For Bayesian analysis, information on the choice of priors and Markov chain Monte Carlo settings
☒	☐	For hierarchical and complex designs, identification of the appropriate level for tests and full reporting of outcomes
☒	☐	Estimates of effect sizes (e.g. Cohen's *d*, Pearson's *r*), indicating how they were calculated

Our web collection on statistics for biologists contains articles on many of the points above.

Software and code

Policy information about availability of computer code

Data collection	no software used
Data analysis	Statistical analysis was performed using Graphpad Prism version 5. Flow cytometry data was analyzed using FlowJo software. Microarray data was analyzed with Transcriptomic Analysis Control (TAC).

For manuscripts utilizing custom algorithms or software that are central to the research but not yet described in published literature, software must be made available to editors and reviewers. We strongly encourage code deposition in a community repository (e.g. GitHub). See the Nature Portfolio guidelines for submitting code & software for further information.

Data

Policy information about availability of data

All manuscripts must include a data availability statement. This statement should provide the following information, where applicable:

- Accession codes, unique identifiers, or web links for publicly available datasets
- A description of any restrictions on data availability
- For clinical datasets or third party data, please ensure that the statement adheres to our policy

Source data are provided with this paper.
The data sets generated in this study are available at Gene Expression Omnibus, available online. Accession numbers: GSE272140 and GSE272043.

Research involving human participants, their data, or biological material

Policy information about studies with human participants or human data. See also policy information about sex, gender (identity/presentation), and sexual orientation and race, ethnicity and racism.

Reporting on sex and gender	not applicable, no reporting on human data
Reporting on race, ethnicity, or other socially relevant groupings	not applicable, no reporting on human data
Population characteristics	not applicable, no reporting on human data
Recruitment	not applicable, no reporting on human data
Ethics oversight	not applicable, no reporting on human data

Note that full information on the approval of the study protocol must also be provided in the manuscript.

Field-specific reporting

Please select the one below that is the best fit for your research. If you are not sure, read the appropriate sections before making your selection.

☒ Life sciences ☐ Behavioural & social sciences ☐ Ecological, evolutionary & environmental sciences

For a reference copy of the document with all sections, see nature.com/documents/nr-reporting-summary-flat.pdf

Life sciences study design

All studies must disclose on these points even when the disclosure is negative.

Sample size	For the in vitro experiments a sample size of at least n=3 was used for accurate statistical analyses. For the in vivo experiments, a sample size of at least n=5 was used ensuring adequate statistcal analyses.
Data exclusions	No data were excluded
Replication	All experiments were independently and reliably repeated three times as indicated in the figures and legends.
Randomization	For the in vivo treatment experiments, mice were randomized before the experimental treatment started. All mice were included into our analyses.
Blinding	The in vivo treatment experiments were performed in a non-blinded manner. The histopathological analyses were conducted by a pathologist in a blinded manner.

Behavioural & social sciences study design

All studies must disclose on these points even when the disclosure is negative.

Study description	not applicable
Research sample	not applicable
Sampling strategy	not applicable
Data collection	not applicable
Timing	not applicable
Data exclusions	not applicable
Non-participation	not applicable
Randomization	not applicable

Ecological, evolutionary & environmental sciences study design

All studies must disclose on these points even when the disclosure is negative.

Study description	not applicable
Research sample	not applicable
Sampling strategy	not applicable
Data collection	not applicable
Timing and spatial scale	not applicable
Data exclusions	not applicable
Reproducibility	not applicable
Randomization	not applicable
Blinding	not applicable

Did the study involve field work? ☐ Yes ☒ No

Reporting for specific materials, systems and methods

We require information from authors about some types of materials, experimental systems and methods used in many studies. Here, indicate whether each material, system or method listed is relevant to your study. If you are not sure if a list item applies to your research, read the appropriate section before selecting a response.

Materials & experimental systems

n/a	Involved in the study	
☐	☒	Antibodies
☐	☒	Eukaryotic cell lines
☒	☐	Palaeontology and archaeology
☐	☒	Animals and other organisms
☒	☐	Clinical data
☒	☐	Dual use research of concern
☒	☐	Plants

Methods

n/a	Involved in the study	
☒	☐	ChIP-seq
☐	☒	Flow cytometry
☒	☐	MRI-based neuroimaging

Antibodies

Antibodies used	STAT5 (G-2) and pJAK2 (21870-R) antibodies, Santa Cruz Biotechnology (Santa Cruz, CA, USA)<br>JAK2 c-terminal antibody (D2E12 XPR) and pSTAT5, Cell Signaling Technology (Danvers, MA, USA)<br>Akt (pan) (11E7), pAkt (Ser473) (D9E), b-Actin (D6A8), HSP90 (4874s), STAT3 (124H6), pSTAT3 (Tyr705) (D3A7), P44/42 (Erk1/2) (127F5), p-p44/42 MAPK (Erk1/2)(Thr202/Tyr204)(D13.14.4E), all Cell Signaling Technology (Danvers, MA, USA)<br>CD45, CD11b, Gr-1, CD 90.2 and B220, all eBioscience<br>TER-119/ CD3/ Gr-1– PE-Cy7 (1:1,000 each), all eBioscience<br>CD31-Pacific Blue (PECAM-1) (1:100), all eBioscience<br>Sca-1-APC-Cy7 (1:100), CD166-PE (ALCAM) (1:50), CD140-PE (1:50), all eBioscience<br>pSTAT5 Alexa fluor647 (Phosflow), P7694, IgG1k Alexa fluor 647 (Phosflow), MPC-21, all eBioscience<br>TNF-a APC, GM-CSF APC, all Biolegend
Validation	All antibodies are validated as per instructions of the manufacturers.

Eukaryotic cell lines

Policy information about cell lines and Sex and Gender in Research

Cell line source(s)	Ba/F3 cells and NIH/3T3 cells were obtained from the German Resource Centre for Biological Material (DSMZ)<br>Phoenix E helper-virus free ecotropic packaging cells were a kind gift from G. Nolan, Stanford, USA.
Authentication	All cell lines mentioned in this study were authenticated at DSMZ,Germany and timely checked for PCR assays with species-

	specific primers.
Mycoplasma contamination	Cells were tested and confirmed mycoplasma free.
Commonly misidentified lines (See ICLAC register)	No commonly misidentified cell lines were used in this study.

Animals and other research organisms

Policy information about studies involving animals; ARRIVE guidelines recommended for reporting animal research, and Sex and Gender in Research

Laboratory animals	Balb/cAan (Janvier Labs, Le Genest-Saint-Isle, France) were kept at the animal facility of the University Hospital Freiburg (University of Freiburg) at 20-24°C with a 12 h light/dark cycle and humidity ranging 45-65%. Mice were housed in individually ventilated cages under specific pathogen-free (SPF) conditions and received acidified and autoclaved water. Mice were used between 8-10 weeks of age.
Wild animals	No wild animas were involved.
Reporting on sex	Male and Female mice were used.
Field-collected samples	The study did not involve field-collected samples.
Ethics oversight	All procedures were reviewd and approved by the University's animal care committee and the local government (Veterinärwesen, Gesundheitlicher Verbraucherschutz und Lebensmittelüberwachung, Regierungspräsidium Freiburg, Freiburg Germany) in Freiburg (protocol number: G-13/05, G-22/093, and G-24/003).

Note that full information on the approval of the study protocol must also be provided in the manuscript.

Plants

Seed stocks	not applicable
Novel plant genotypes	not applicable
Authentication	not applicable

Flow Cytometry

Plots

Confirm that:

- [x] The axis labels state the marker and fluorochrome used (e.g. CD4-FITC).
- [x] The axis scales are clearly visible. Include numbers along axes only for bottom left plot of group (a 'group' is an analysis of identical markers).
- [x] All plots are contour plots with outliers or pseudocolor plots.
- [x] A numerical value for number of cells or percentage (with statistics) is provided.

Methodology

Sample preparation	Cells from freshly isolated mouse peripheral blood, bone marrow and spleen were analysed by flow cytometry. Cells are harvested based on cell type and counted using haemocytometer. Cell viability was analysed using 7-AAD (BD Biosciences), 20 min incubation on ice. Cells were washed with FACS buffer two times. For mouse PBMC, spleen or bone marrow, FC receptor blokade (1:50) (BD Biosciences, germany) was used for 15-20 min on ice before staining. Cells were washed twice with ice-cold 1x PBS after indicated stainings and re-suspended in 250ul/mL 1x PBS. For stromal niche cell isolation, flushed femurs and tibias were crushed with mortar and pestle. Bone chips were washed several times in PBS until the chips were white. Endosteal stromal cells were released from the hematopoietic-depleted bone chips by digestion with 3 mg/mL collagenase type I, 0.5 mg/ml collagenase Type II, and 15 µg/mL DNAse dissolved in PBS for 1 hour at 37°C at 110 rpm. The stromal and the BM fraction were used in all subsequent analyses. For bone chips cells, we used CD45/ TER-119/ CD3/ Gr-1/ (lineage) – PE-Cy7 (1:1,000 each), CD31-Pacific Blue (PECAM-1) (1:100), Sca-1-APC-Cy7 (1:100), CD166-PE (ALCAM) (1:50). For the BM fraction we used CD45/ TER-119/ CD3/ Gr-1/ (lineage markers) – PE-Cy7 (1:1,000 each) CD31-Pacific Blue (1:100), Sca-1-APC-Cy7 (1:100), CD140-PE (1:50). The stained cells were analyzed by FACS Vantage (BD Biosciences).

	Cells from spleen, bone marrow and PB were isolated after erythrocytes lysis followed by centrifugation.
Instrument	Flow cytometry data were acquired on a FACS Vantage or BD LSR Fortessa. Sorting of mesenchymal stromal cells and endothelial were performed on BD FACSAria™ III.
Software	FlowJo
Cell population abundance	Numbers of transduced enhanced green fluorescent protein (EGFP) or enhanced yellow fluorescent protein (EYFP) positive cells in the peripheral blood of transplanted mice were determined by flow cytometry. Unstained or EGFP/EYFP negative cells, single stains and fluorescence minus one (FMO) controls were utilized.
Gating strategy	Appropriate controls were included in all experiments. Live cells are gated by FCS-A vs. SSC-A, followed by doublets exclusion. For myeloid cell population of peripheral blood, spleen and bone marrow were gated by FITC-CD11b against EGFP. For the percentage of granulocytes PE-Gr-1 against EGFP, for lymphoid populations B220-APC, and Thy1.2-BV510 against EGFP. Unstained controls, single stains and fluorescence minus one (FMO) controls were used for gating on right parameters and to check between the positive and negative cells. Gating strategy will be provided upon request.

☒ Tick this box to confirm that a figure exemplifying the gating strategy is provided in the Supplementary Information.
