# Supplementary material for: Efficacy of JAK1/2 inhibition in murine myeloproliferative neoplasms is not mediated by targeting oncogenic signaling
Source: Nat Commun. 2025 May 24;16:4833. doi: 10.1038/s41467-025-60019-6 (PMC12103521; doi:10.1038/s41467-025-60019-6)
Supplement: Supplementary file 4 — Source Data [file 41467_2025_60019_MOESM4_ESM.zip › Gorantla et al., NCOMMS-24-38297B, Source Data, Raw images of western blots, 2025-5-4.pptx]

## Slide 1
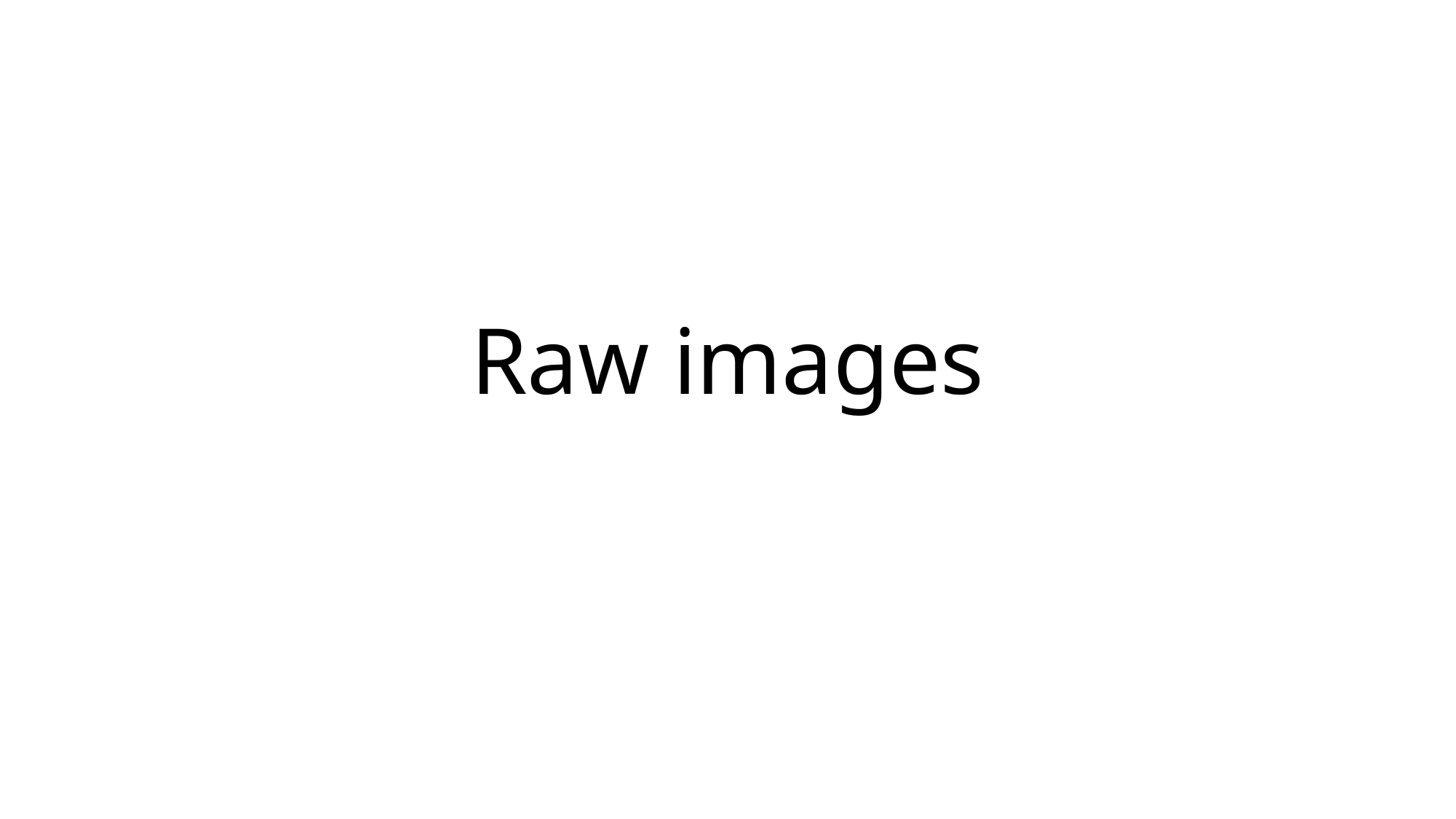

# Raw images

## Slide 2
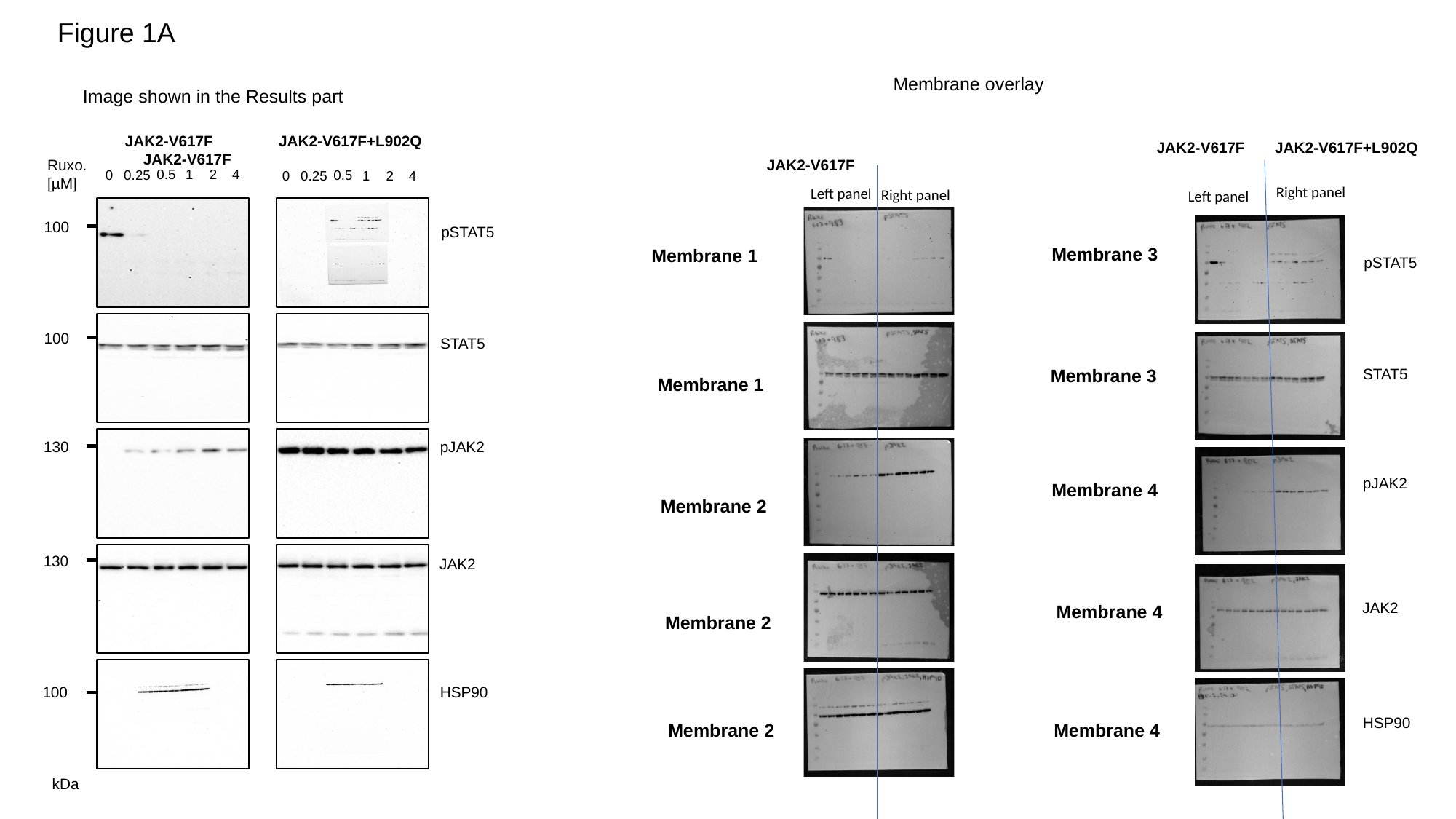

Figure 1A
Membrane overlay
Image shown in the Results part
JAK2-V617F
JAK2-V617F+L902Q
JAK2-V617F+L902Q
JAK2-V617F
JAK2-V617F
Ruxo.
[µM]
JAK2-V617F
Left panel
Right panel
Membrane 1
Membrane 1
Membrane 2
Membrane 2
Membrane 2
0.5
1
2
4
0
0.25
0.5
1
2
4
0
0.25
Right panel
Left panel
100
pSTAT5
Membrane 3
pSTAT5
100
STAT5
Membrane 3
STAT5
130
pJAK2
pJAK2
Membrane 4
130
JAK2
JAK2
Membrane 4
HSP90
100
HSP90
Membrane 4
kDa

## Slide 3
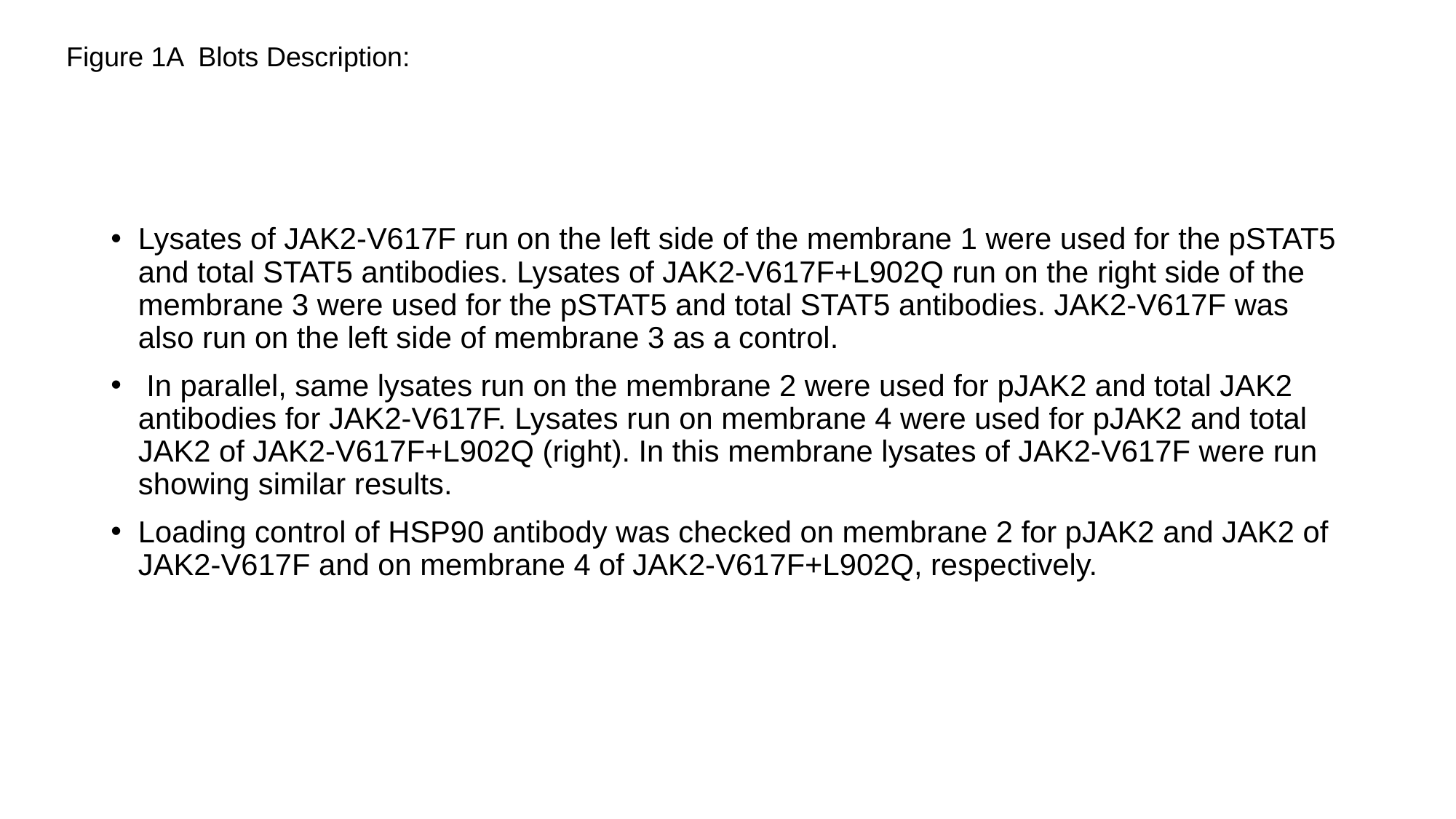

Figure 1A Blots Description:
Lysates of JAK2-V617F run on the left side of the membrane 1 were used for the pSTAT5 and total STAT5 antibodies. Lysates of JAK2-V617F+L902Q run on the right side of the membrane 3 were used for the pSTAT5 and total STAT5 antibodies. JAK2-V617F was also run on the left side of membrane 3 as a control.
 In parallel, same lysates run on the membrane 2 were used for pJAK2 and total JAK2 antibodies for JAK2-V617F. Lysates run on membrane 4 were used for pJAK2 and total JAK2 of JAK2-V617F+L902Q (right). In this membrane lysates of JAK2-V617F were run showing similar results.
Loading control of HSP90 antibody was checked on membrane 2 for pJAK2 and JAK2 of JAK2-V617F and on membrane 4 of JAK2-V617F+L902Q, respectively.

## Slide 4
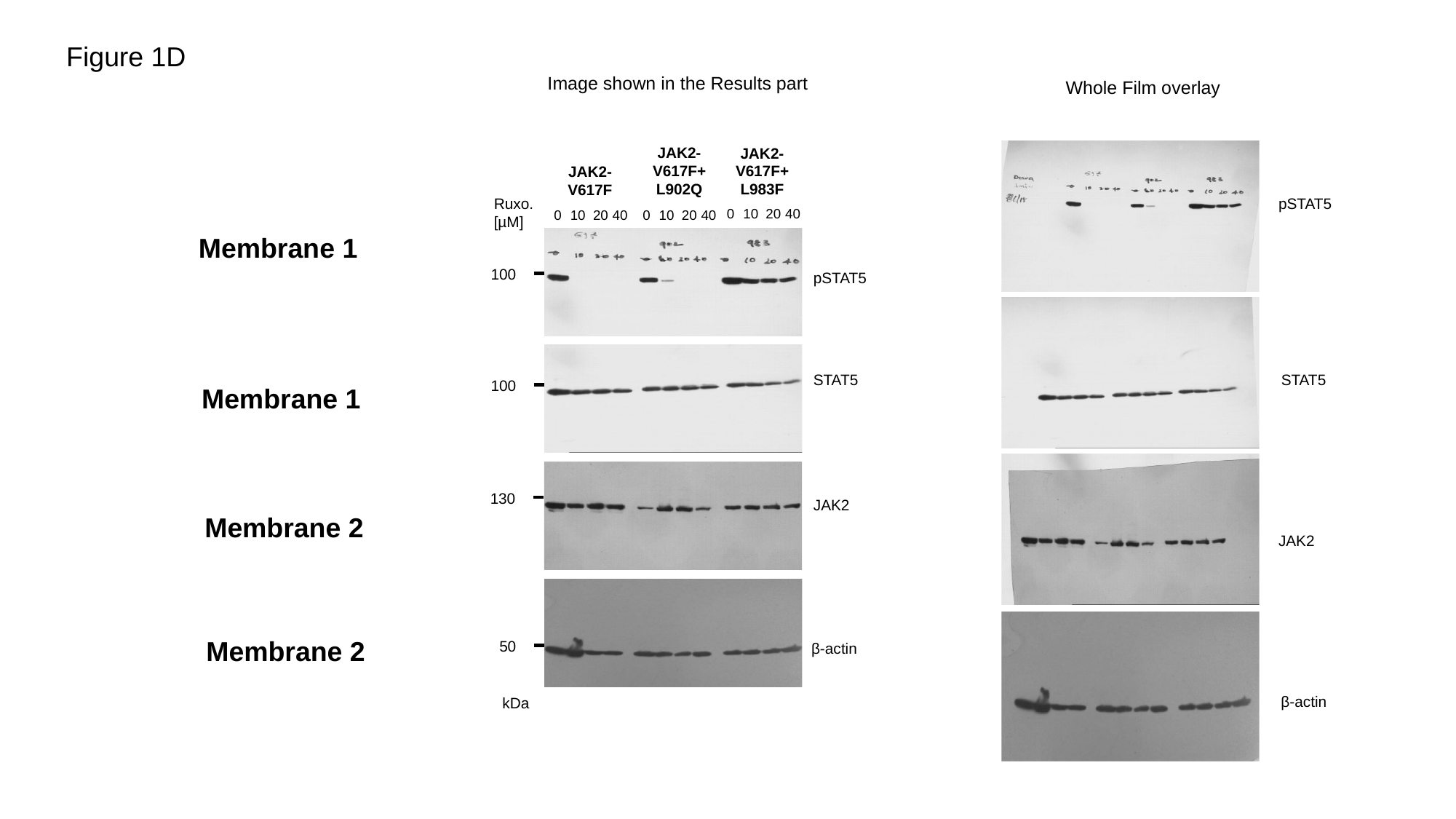

Figure 1D
Image shown in the Results part
Whole Film overlay
JAK2-
V617F+
L902Q
JAK2-
V617F+
L983F
JAK2-
V617F
Ruxo.
[µM]
pSTAT5
10
20
40
0
10
20
40
10
20
40
0
0
Membrane 1
100
pSTAT5
STAT5
STAT5
100
Membrane 1
130
JAK2
Membrane 2
JAK2
Membrane 2
50
β-actin
β-actin
kDa

## Slide 5
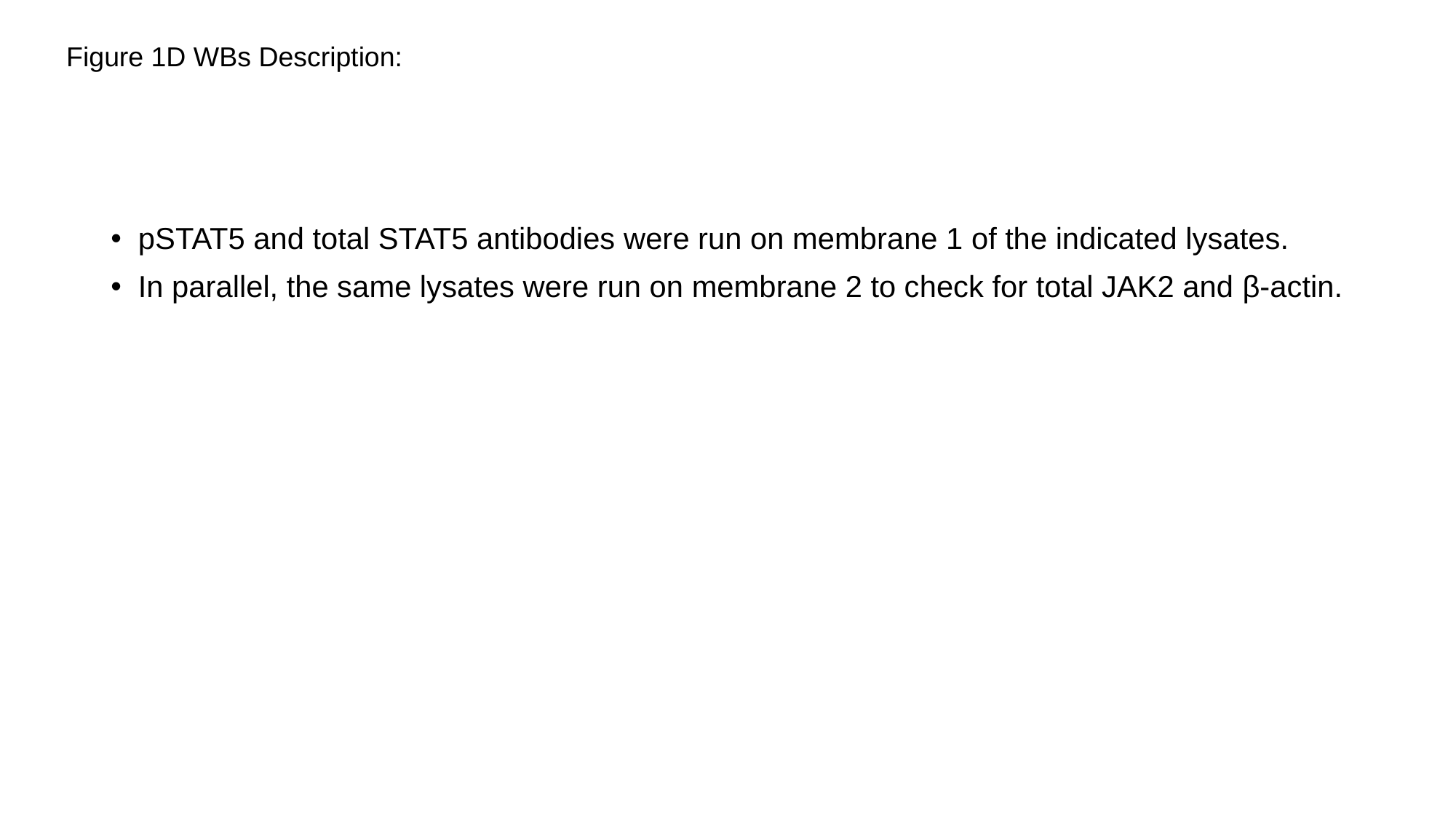

Figure 1D WBs Description:
pSTAT5 and total STAT5 antibodies were run on membrane 1 of the indicated lysates.
In parallel, the same lysates were run on membrane 2 to check for total JAK2 and β-actin.

## Slide 6
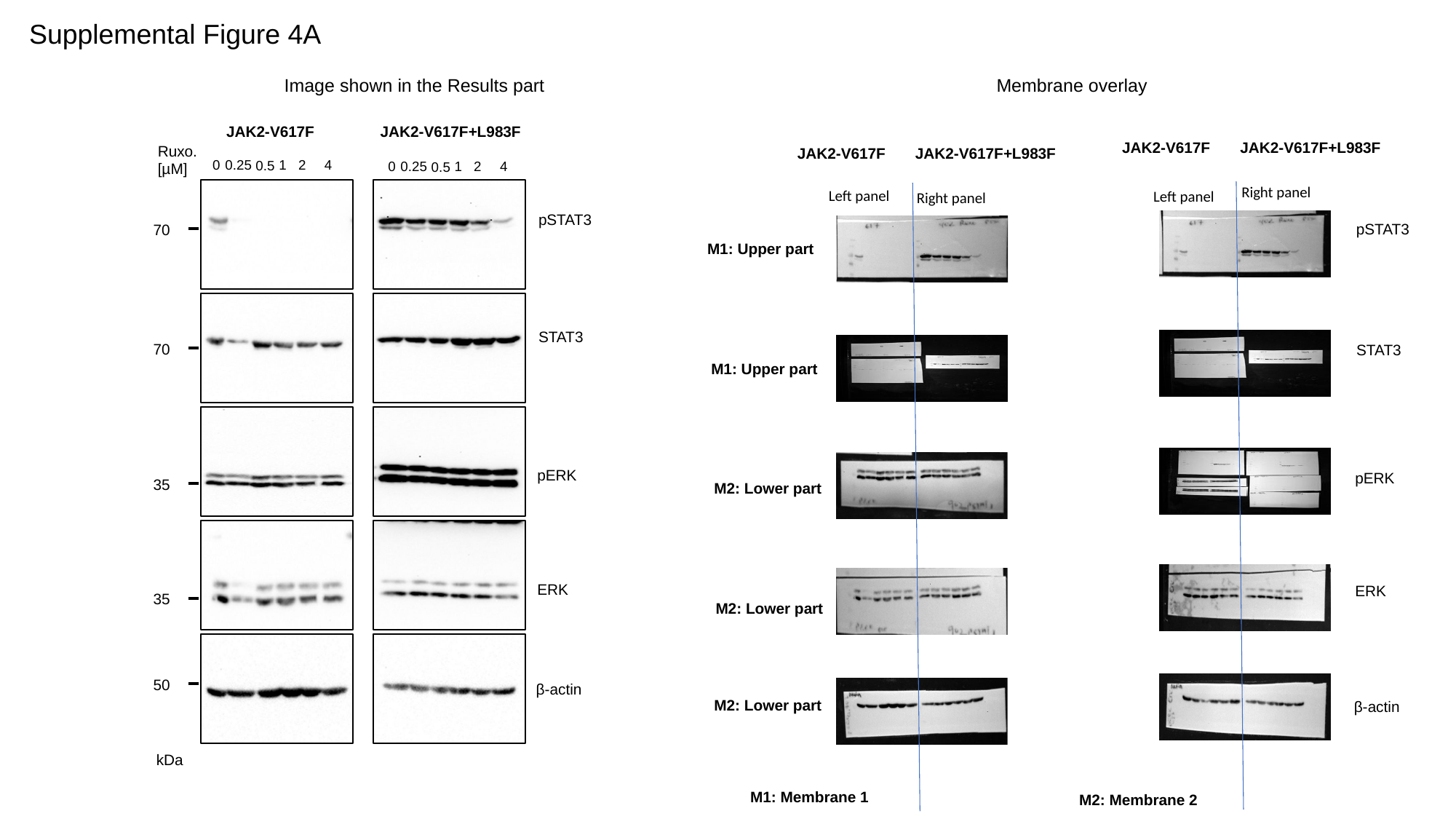

Supplemental Figure 4A
Image shown in the Results part
Membrane overlay
JAK2-V617F+L983F
1
2
4
0
0.25
0.5
pSTAT3
STAT3
pERK
ERK
β-actin
JAK2-V617F
JAK2-V617F+L983F
JAK2-V617F
Ruxo.
[µM]
JAK2-V617F+L983F
JAK2-V617F
1
2
4
0
0.25
0.5
Right panel
Left panel
Left panel
Right panel
pSTAT3
70
M1: Upper part
70
STAT3
M1: Upper part
pERK
35
M2: Lower part
ERK
35
M2: Lower part
50
M2: Lower part
β-actin
kDa
M1: Membrane 1
M2: Membrane 2

## Slide 7
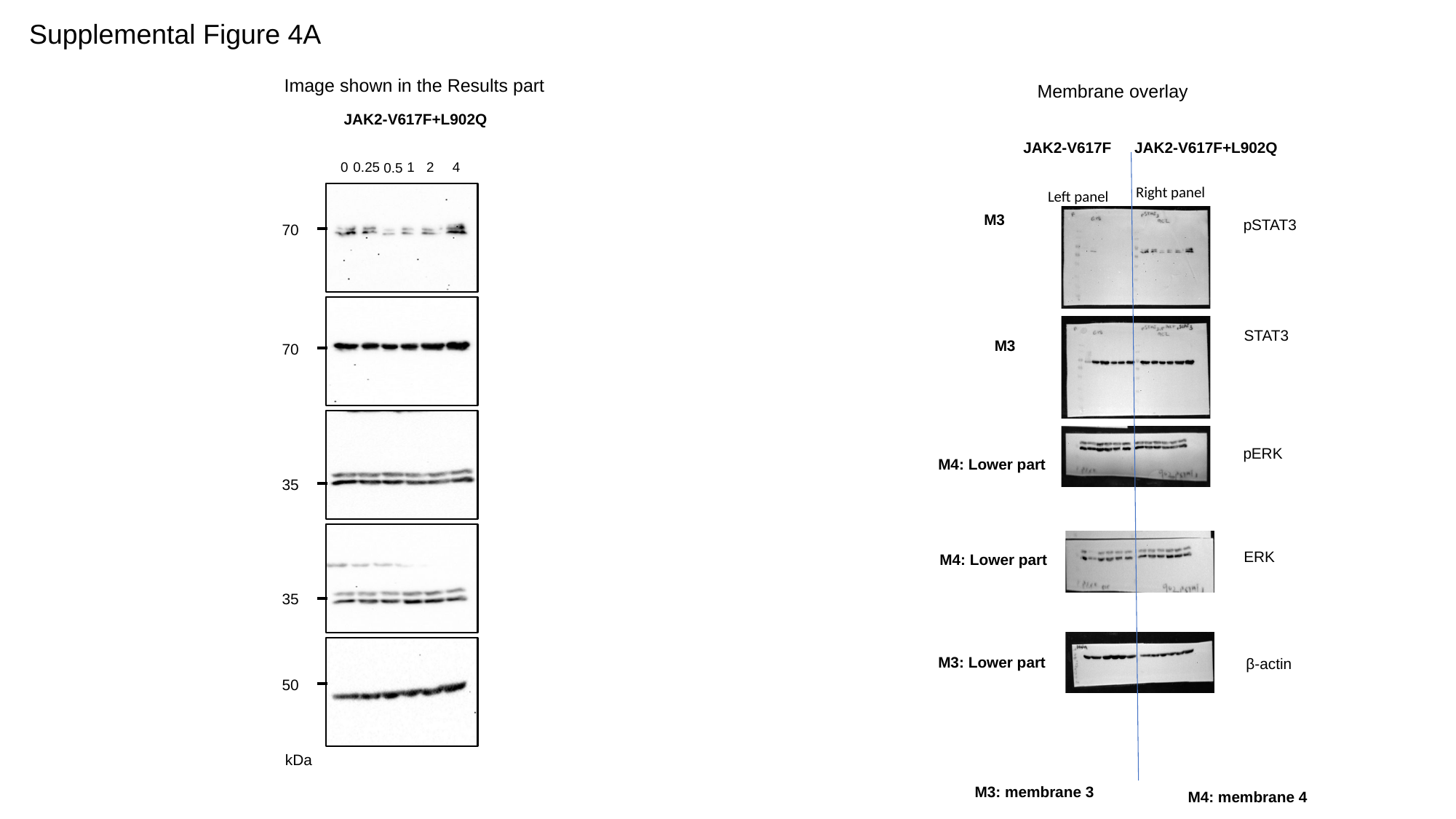

Supplemental Figure 4A
Image shown in the Results part
Membrane overlay
JAK2-V617F+L902Q
JAK2-V617F+L902Q
JAK2-V617F
1
2
4
0
0.25
0.5
Right panel
Left panel
M3
pSTAT3
70
STAT3
M3
70
pERK
M4: Lower part
35
ERK
M4: Lower part
35
M3: Lower part
β-actin
50
kDa
M3: membrane 3
M4: membrane 4

## Slide 8
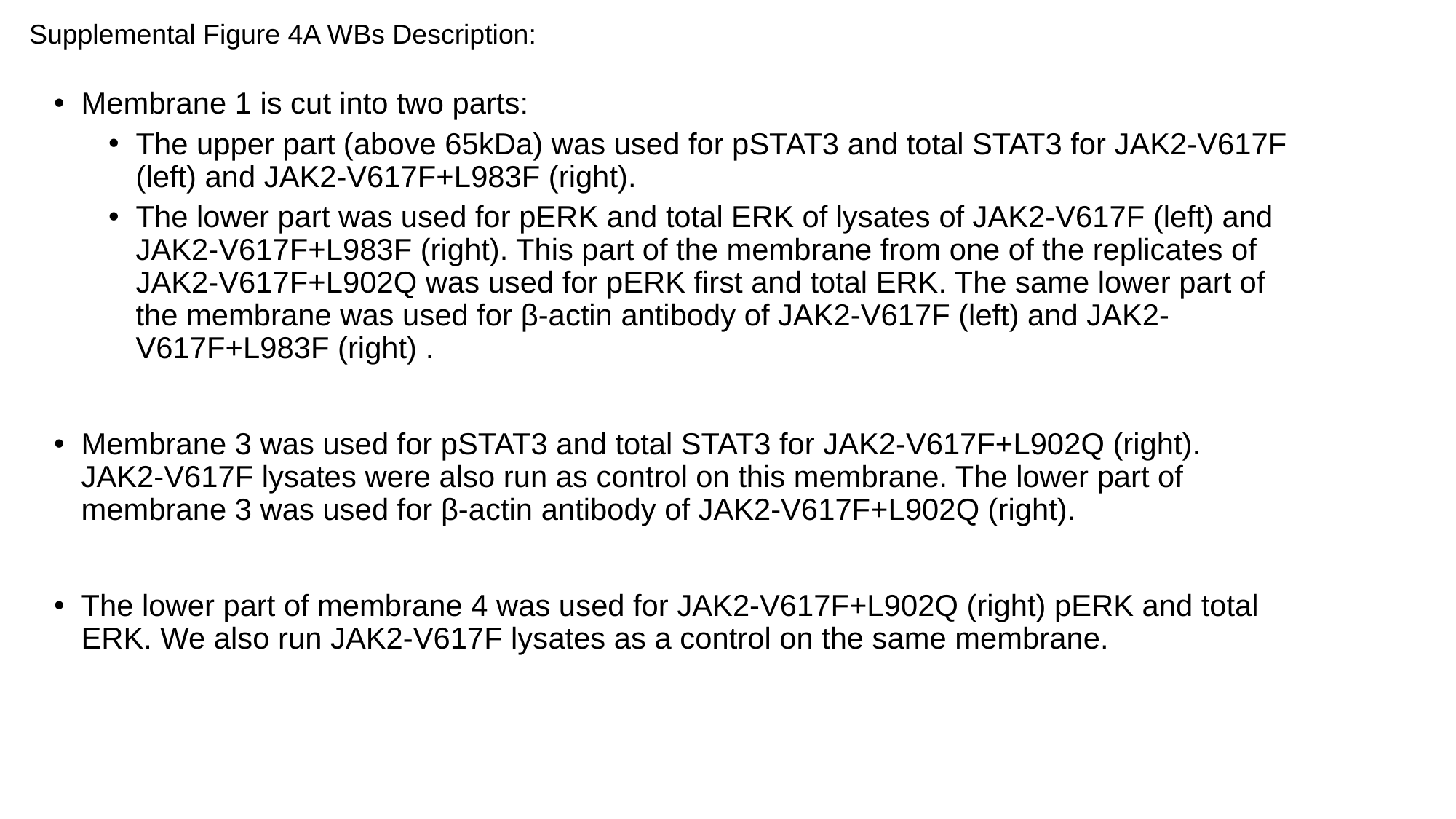

Supplemental Figure 4A WBs Description:
Membrane 1 is cut into two parts:
The upper part (above 65kDa) was used for pSTAT3 and total STAT3 for JAK2-V617F (left) and JAK2-V617F+L983F (right).
The lower part was used for pERK and total ERK of lysates of JAK2-V617F (left) and JAK2-V617F+L983F (right). This part of the membrane from one of the replicates of JAK2-V617F+L902Q was used for pERK first and total ERK. The same lower part of the membrane was used for β-actin antibody of JAK2-V617F (left) and JAK2-V617F+L983F (right) .
Membrane 3 was used for pSTAT3 and total STAT3 for JAK2-V617F+L902Q (right). JAK2-V617F lysates were also run as control on this membrane. The lower part of membrane 3 was used for β-actin antibody of JAK2-V617F+L902Q (right).
The lower part of membrane 4 was used for JAK2-V617F+L902Q (right) pERK and total ERK. We also run JAK2-V617F lysates as a control on the same membrane.

## Slide 9
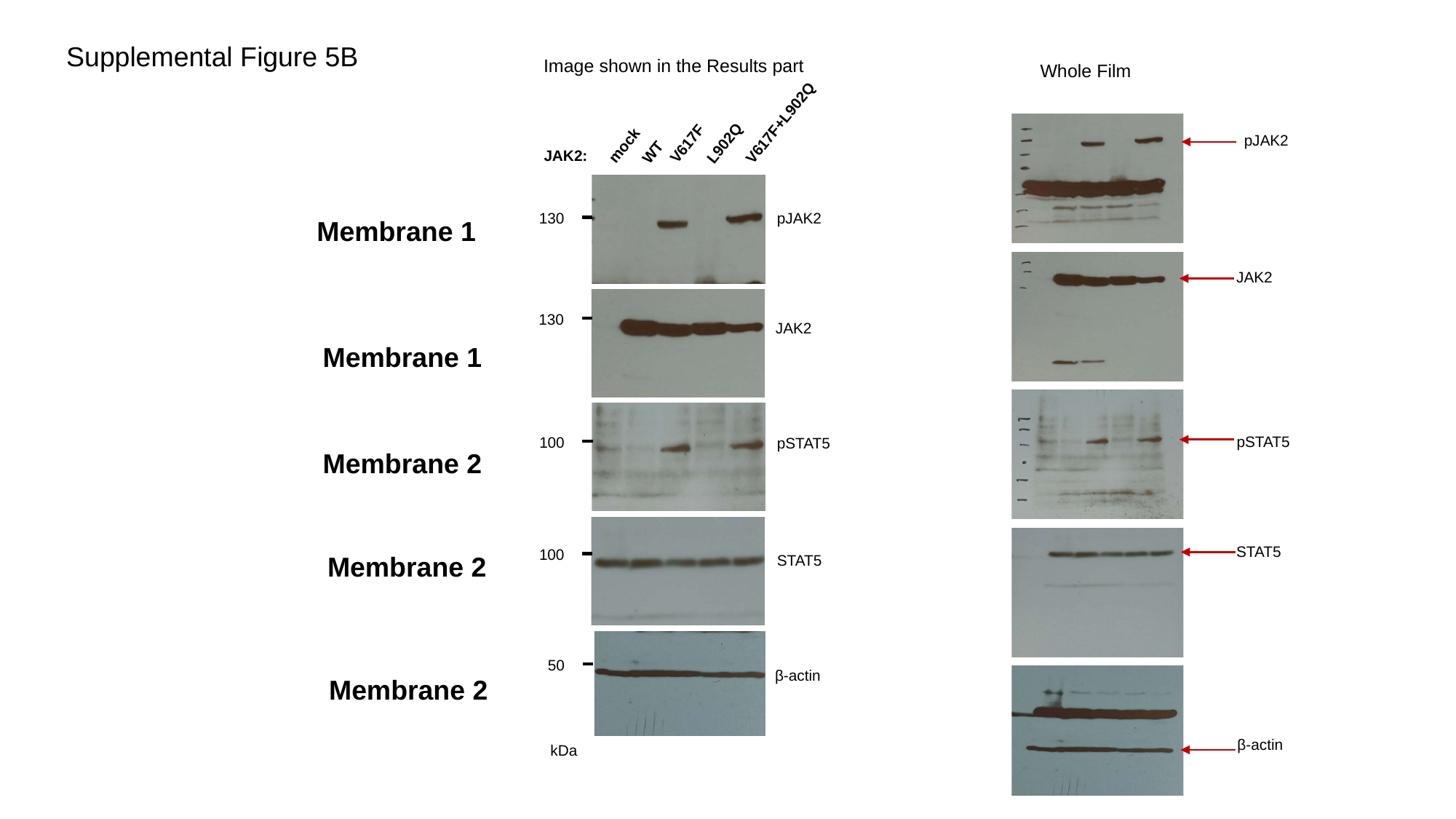

Supplemental Figure 5B
Image shown in the Results part
Whole Film
V617F+L902Q
pJAK2
L902Q
V617F
mock
WT
JAK2:
pJAK2
130
Membrane 1
JAK2
130
JAK2
Membrane 1
pSTAT5
100
pSTAT5
Membrane 2
STAT5
100
Membrane 2
STAT5
50
β-actin
Membrane 2
β-actin
kDa

## Slide 10
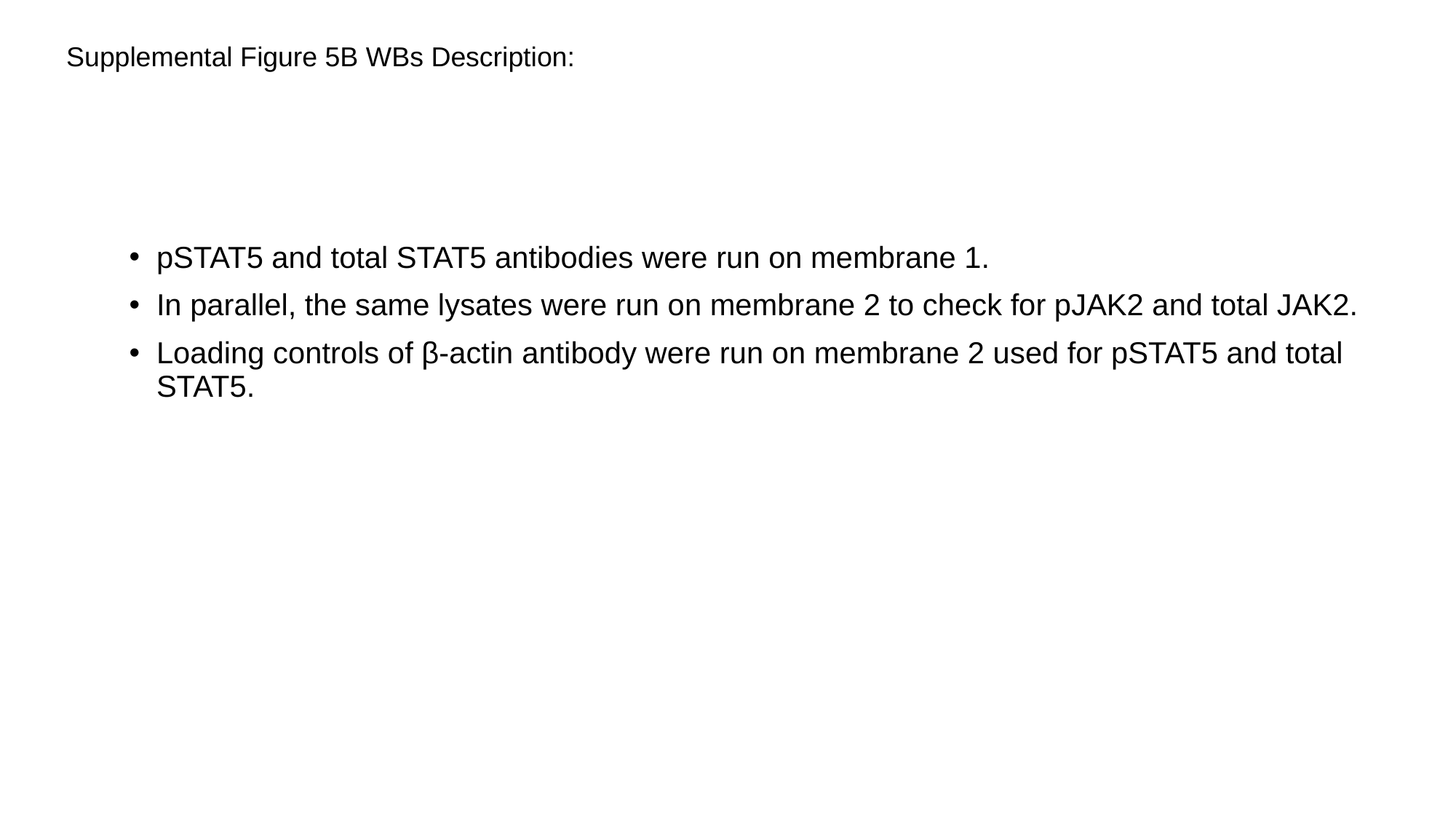

Supplemental Figure 5B WBs Description:
pSTAT5 and total STAT5 antibodies were run on membrane 1.
In parallel, the same lysates were run on membrane 2 to check for pJAK2 and total JAK2.
Loading controls of β-actin antibody were run on membrane 2 used for pSTAT5 and total STAT5.

## Slide 11
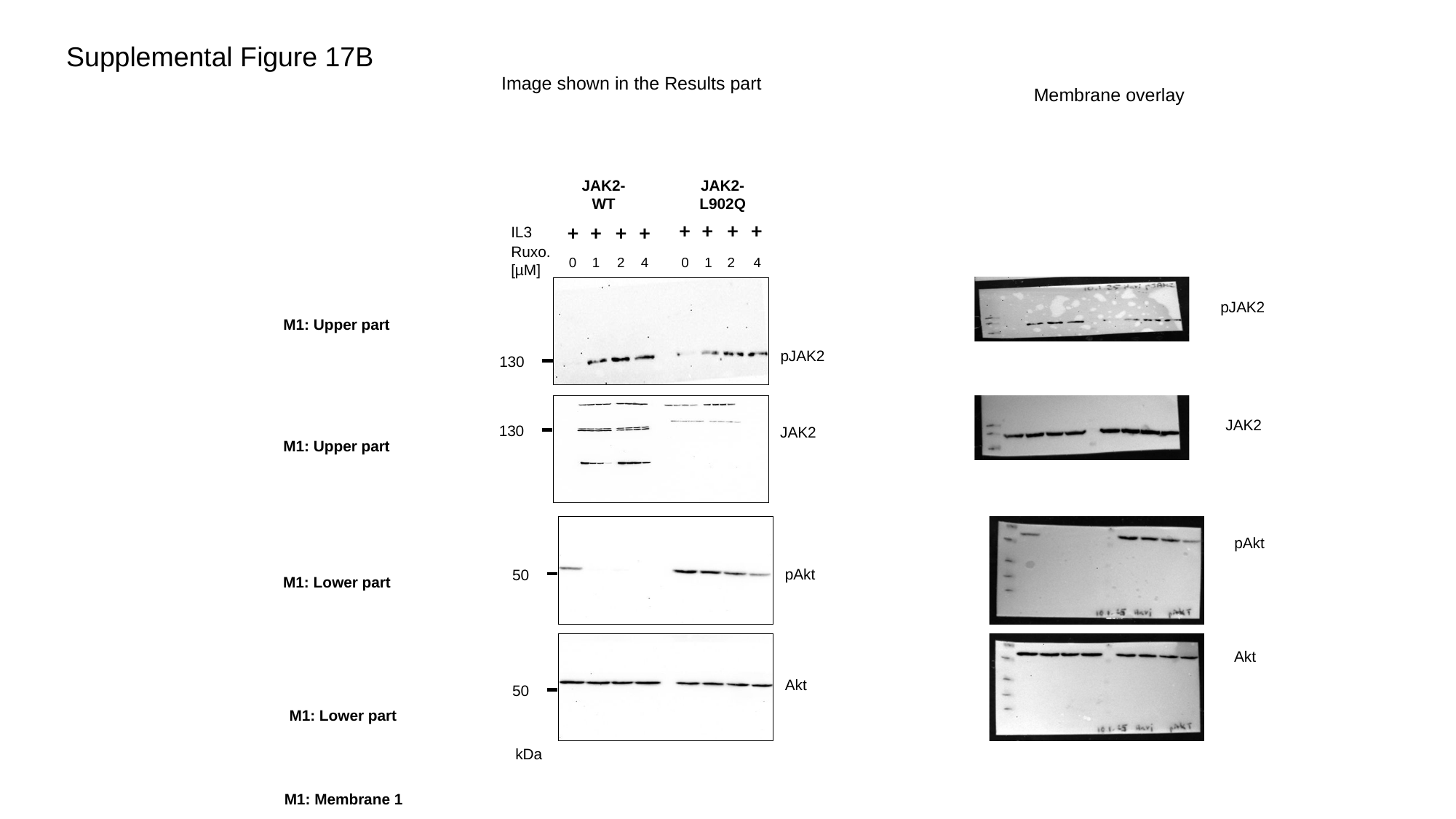

Supplemental Figure 17B
Image shown in the Results part
Membrane overlay
JAK2-
WT
JAK2-
L902Q
+
+
+
+
+
+
+
+
IL3
Ruxo.
[µM]
1
2
4
1
2
4
0
0
pJAK2
M1: Upper part
pJAK2
130
JAK2
130
JAK2
M1: Upper part
pAkt
pAkt
50
M1: Lower part
Akt
Akt
50
M1: Lower part
kDa
M1: Membrane 1

## Slide 12
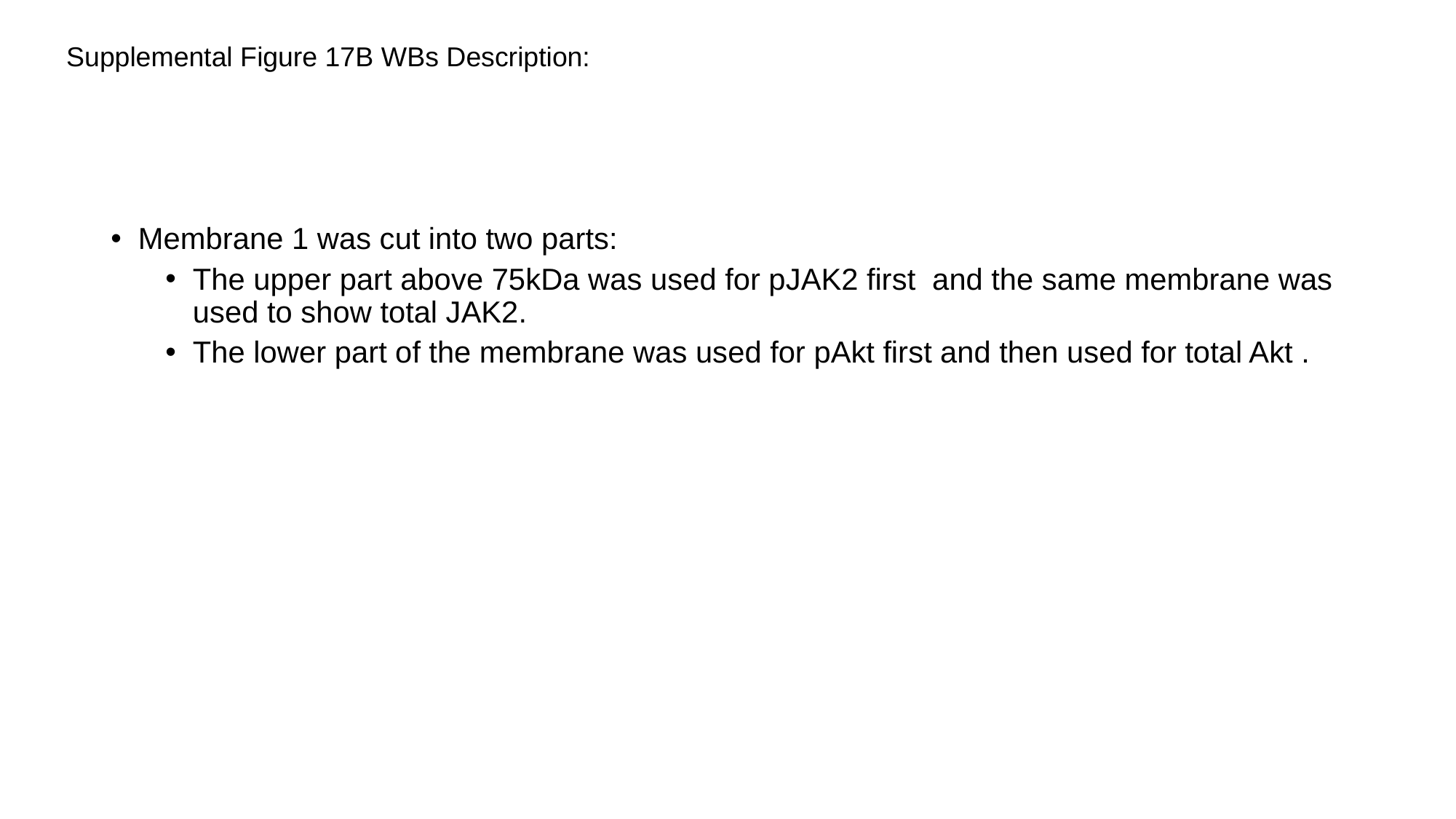

Supplemental Figure 17B WBs Description:
Membrane 1 was cut into two parts:
The upper part above 75kDa was used for pJAK2 first and the same membrane was used to show total JAK2.
The lower part of the membrane was used for pAkt first and then used for total Akt .

## Slide 13
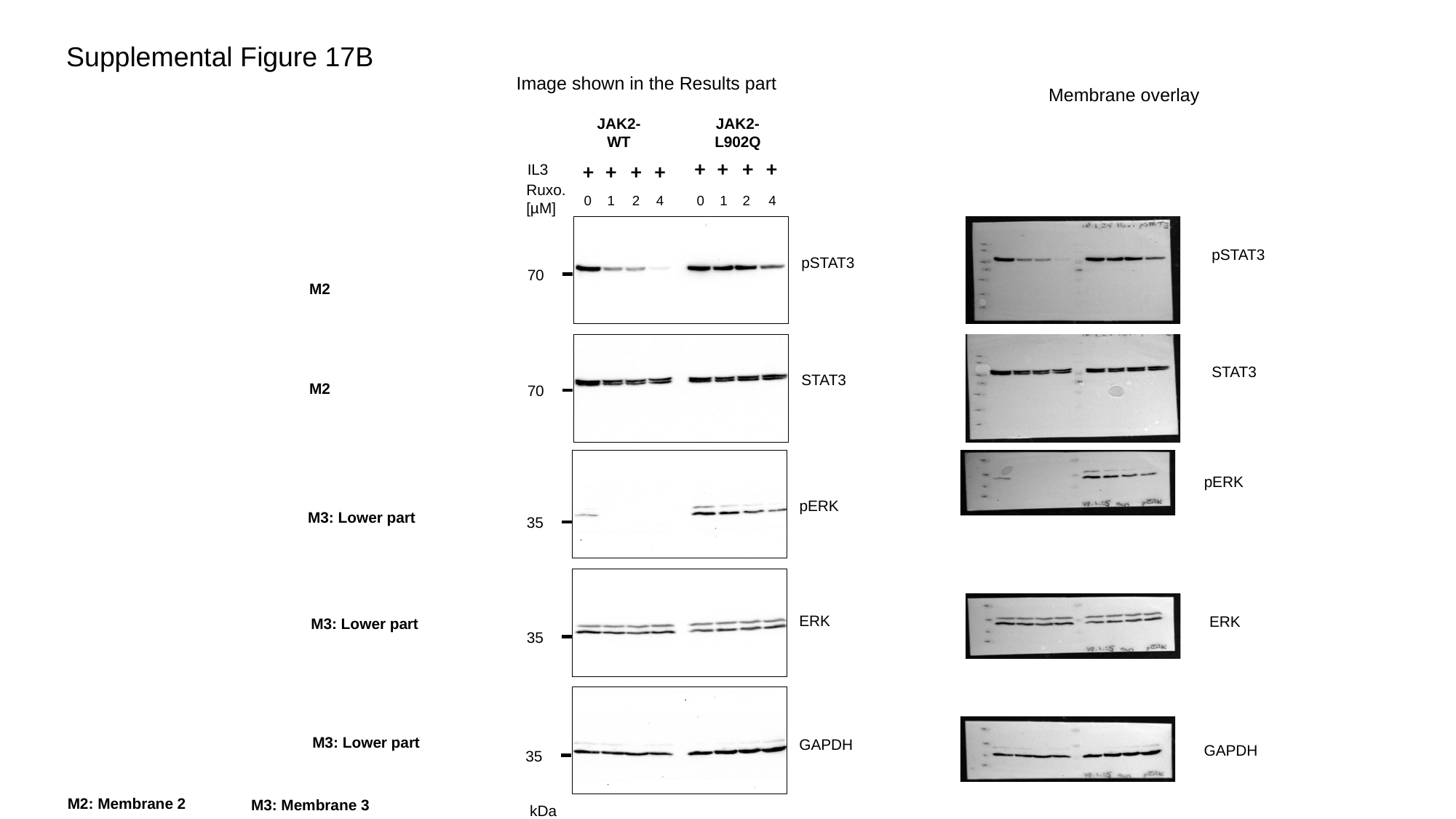

Supplemental Figure 17B
Image shown in the Results part
Membrane overlay
JAK2-
WT
JAK2-
L902Q
+
+
+
+
+
+
+
+
IL3
Ruxo.
[µM]
1
2
4
1
2
4
0
0
pSTAT3
pSTAT3
70
M2
STAT3
STAT3
M2
70
pERK
pERK
M3: Lower part
35
ERK
ERK
M3: Lower part
35
M3: Lower part
GAPDH
GAPDH
35
M2: Membrane 2
M3: Membrane 3
kDa

## Slide 14
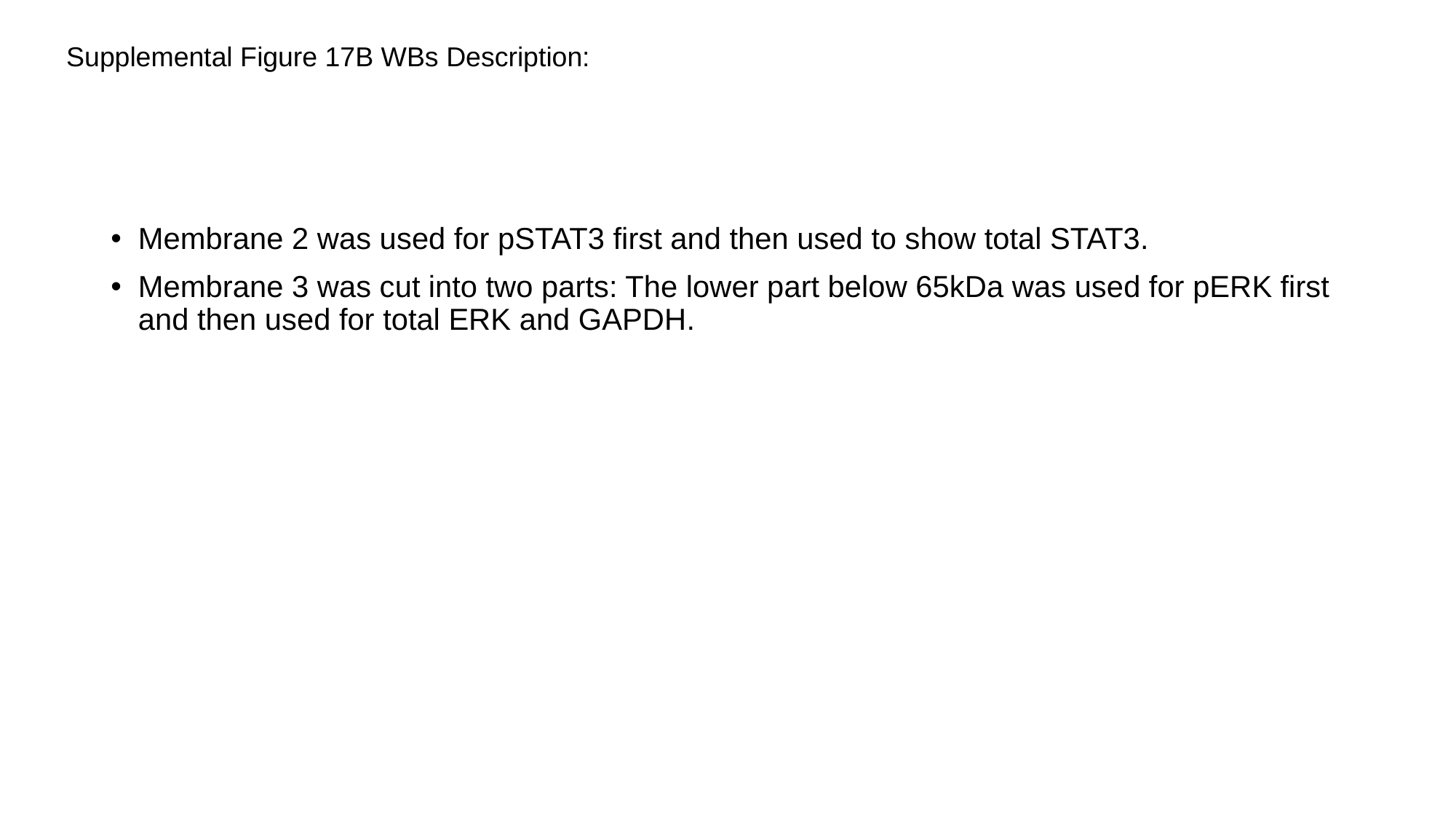

Supplemental Figure 17B WBs Description:
Membrane 2 was used for pSTAT3 first and then used to show total STAT3.
Membrane 3 was cut into two parts: The lower part below 65kDa was used for pERK first and then used for total ERK and GAPDH.
